# A comprehensive review on sustainable clay-based geopolymers for wastewater treatment: circular economy and future outlook

**DOI:** 10.1007/s10661-023-11303-9

**Published:** 2023-05-19

**Authors:** Ali Maged, Hadeer Abd El-Fattah, Rasha M. Kamel, Sherif Kharbish, Ahmed M. Elgarahy

**Affiliations:** 1 Geology Department, Faculty of Science, Suez University, P.O. Box 43518, El Salam City, Suez Governorate Egypt; 2 Chemistry Department, Faculty of Science, Suez University, P.O. Box 43518, El Salam City, Suez Governorate Egypt; 3 Egyptian Propylene and Polypropylene Company (EPPC), Port-Said, Egypt; 4 Environmental Chemistry Division, Environmental Science Department, Faculty of Science, Port Said University, Port Said, Egypt

**Keywords:** Geopolymers, Clays, Water treatment, Adsorption mechanisms, Clay-based geopolymers, Circular economy

## Abstract

In the present era of significant industrial development, the presence and dispersal of countless water contaminants in water bodies worldwide have rendered them unsuitable for various forms of life. Recently, the awareness of environmental sustainability for wastewater treatment has increased rapidly in quest of meeting the global water demand. Despite numerous conventional adsorbents on deck, exploring low-cost and efficient adsorbents is interesting. Clays and clays-based geopolymers are intensively used as natural, alternative, and promising adsorbents to meet the goals for combating climate change and providing low carbon, heat, and power. In this narrative work, the present review highlights the persistence of some inorganic/organic water pollutants in aquatic bodies. Moreover, it comprehensively summarizes the advancement in the strategies associated with synthesizing clays and their based geopolymers, characterization techniques, and applications in water treatment. Furthermore, the critical challenges, opportunities, and future prospective regarding the circular economy are additionally outlined. This review expounded on the ongoing research studies for leveraging these eco-friendly materials to address water decontamination. The adsorption mechanisms of clays-based geopolymers are successfully presented. Therefore, the present review is believed to deepen insights into wastewater treatment using clays and clays-based geopolymers as a groundbreaking aspect in accord with the waste-to-wealth concept toward broader sustainable development goals.

**Supplementary Information:**

The online version contains supplementary material available at 10.1007/s10661-023-11303-9.

## Introduction

**Fig. 1:**
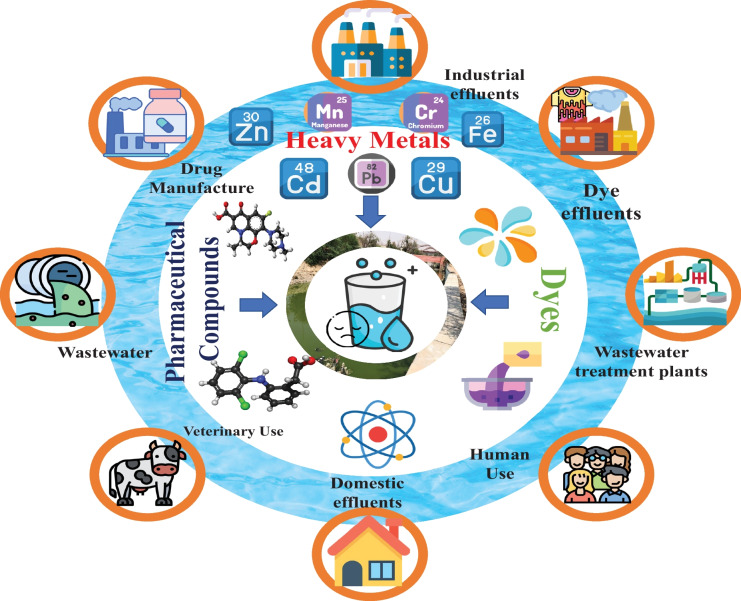
Schematic representation of the most existed pollutants in the aquatic environment

Water is an extraordinarily vital ecological source for natural life, ecosystems, and human society (Dalstein & Naqvi, 2022). One of humanity’s most urgent issues in the twenty-first century is the continuously increasing and unsustainable demand for water worldwide. Therefore, safe and clean water access is integrated into the 2030 Agenda for Sustainable Development Goals (SDGs). The loss of crucial freshwater reserves, increased population, and living requirements are all factors boosting the demand for new water supplies (Yang et al., 2021). Furthermore, 80% of contaminated water is discharged into the environment untreated, resulting in water quality degradation (Chen et al., 2020). Water pollution has become a global problem. According to the World Health Organization records (WHO, 2022), an estimated 829,000 fatalities occur yearly due to inadequate drinking water and sanitation (The World Counts, 2023). According to the world count 2023, approximately 3.57 million deaths arise from water-related diseases. The majority of these casualties are children (2.2 million) (Counts, 2023). The primary cause of water pollution is industrial effluents, wastewater treatment plants, and domestic effluents (Maged et al., 2021). Heavy metals, herbicides, dyes, pesticides, pharmaceuticals, and organic (aromatic) compounds are among the contaminants found in industrial wastewater (Price & Heberling, 2018). These contaminants pose a significant threat to the environment. Figure 1 represents of the most existed pollutants in the aquatic environment with various contamination scenarios.

Heavy metals are naturally poisonous, causing major health problems in humans and animals even at extremely low concentrations (Rasaki et al., 2019). The non-biodegradable nature of heavy metals leads to their bioaccumulation over time, increasing their engagement in living organisms (Maged et al., 2020b). Heavy metal ions are also carcinogenic and can induce organ damage to the lungs, kidneys, liver, prostate, esophagus, stomach, and skin at even minimum concentrations (Kumar et al., 2021). Over thousands-types of synthetic dyes and pigments are currently in commercial production, with an annual output exceeding 7 million tons worldwide. These dyes are used in various industries, such as textiles, carpeting, plastics, leather, paper, printing, foodstuff, and pharmaceuticals (Tran et al., 2023). However, the dye-containing wastewater discharged from these industries can threaten aquatic ecosystems and human health by causing water pollution (Tran et al., 2019). The presence of dyes in water sources hinders sunlight transmission and adversely influences the photosynthesis of aquatic flora. Additionally, most synthetic dyes are toxic, non-biodegradable, and carcinogenic (Elgarahy et al., 2023; Uddin et al., 2021).

Pharmaceutical compounds are chemically stable compounds produced to enhance human health (Abd El-Fattah et al., 2023). Recently, the existence of such compounds in minimal environmental concentrations has been an emergent problem for environmental maintenance (Maged et al., 2020a). Pharmaceutical compounds can be found in the environment through the domestic, industrial, hospital, and livestock farm wastewater, as shown in Fig. 1. These compounds can also be found in sewage treatment plants through the excretion of urine as well as unused and expired pharmaceuticals into sinks (Korkmaz et al., 2022). The presence of pharmaceutical substances in water adversely impacts human health and living ecosystems because they may lead to antibiotic-tolerant bacteria and genetic resistance factors in the marine ecosystem (Maged et al., 2020c). Their content in raw domestic wastewater is demonstrated to be 100.0 ng/L to 10.0 mg/L, but it may attain up to 100–500 mg/L in hospital and pharmaceutical company wastes (Morales-Paredes et al., 2022). Table 1 illustrates some pharmaceutical compounds found in surface, effluent, and portable water in different areas worldwide. Due to the spreading of the COVID-19 pandemic, most antiparasitics, antiprotozoals, antibiotics, glucocorticoids, and antivirals were consumed in large quantities in this virus treatment. Morales-Paredes et al. (2022) reported that the concentration of antiviral agent drugs increased by more than 70% in urban wastewater during the pandemic compared with their concentration before the pandemic. Figure S1 shows the existence of the most used drugs for COVID-19 in different sources (domestic wastewater and surface water) (Morales-Paredes et al., 2022).

**Table 1:** The most detected pharmaceutical compounds, as emerging contaminants in the aquatic environment

Type	Compounds	Environmental occurrence	References
Acetylsalicylic acid	Anti-inflammatory analgesics	Effluent and drinking water	(Biel-Maeso et al., 2018)
	Ibuprofen	Surface, drinking water, river, and effluent	(Biel-Maeso et al., 2018)
	Paracetamol (acetaminophen)	Hospital effluent and drinking water	(Papageorgiou et al., 2019)
	Diclofenac	Effluent and drinking water	(Biel-Maeso et al., 2018)
	Naproxen	Effluent and drinking water	(Biel-Maeso et al., 2018)
	Ketoprofen	Effluent and drinking water	(Biel-Maeso et al., 2018)
Antibiotics	Ciprofloxacin	Effluent and drinking water	(Mahmood et al., 2019)
	Sulfamethoxazole	Effluent and drinking water	(Biel-Maeso et al., 2018; Papageorgiou et al., 2019)
	Erythromycin	Effluent and drinking water	(Biel-Maeso et al., 2018)
	Ofloxacin	Surface, drinking water, river, and effluent	(Biel-Maeso et al., 2018)
	Levofloxacin	Effluent	(Mahmood et al., 2019)
	Metronidazole	Surface, drinking water, river, and sewage	(de Ilurdoz et al., 2022)
	Norfloxacin	Surface, river, and sewage	(de Ilurdoz et al., 2022)
	Trimethoprim	Surface, drinking water, river, sewage and hospital effluent	(de Ilurdoz et al., 2022)
Antineoplastic	Oxaliplatin	Predicted effluent	(Rowney et al., 2011)
	Cisplatin	Predicted effluent	(Rowney et al., 2011)
	5-fluorouracil (5-FU)	Hospital Influent	(Wormington et al., 2020)
Antidiabetic	Metformin	Hospital effluent	(Papageorgiou et al., 2019)
Psychiatric drugs	Carbamazepine	Surface, drinking water, river, and effluent	(Al Aukidy et al., 2012)
Lipid regulators/anti-hypertensives	Fenofibrate	Effluent	(Biel-Maeso et al., 2018)
	Atenolol	Effluent	(Biel-Maeso et al., 2018)
Antivirals	Favipiravir	Surface water	(Azuma et al., 2017)
	Lopinavir	Surface water, domestic water	(Abafe et al., 2018; Wood et al., 2015)
Antiparasitics	Ivermectin	Surface water, hospital wastewater	(Aydin et al., 2019; Rodriguez-Gil et al., 2013)
Antiprotozoals	Chloroquine	Surface water, ground water	(Olatunde et al., 2014)

Various water treatment techniques have been applied to the existing contaminants, such as electrochemical osmosis, flocculation–coagulation, precipitation, and adsorption approaches (Maged et al., 2020a). The adsorption technique has been considered the most applied approach for eradicating inorganic and organic pollutants from polluted waters owing to its facility, performance, and cost-effectiveness. Many researchers have used adsorbent substances such as fly ash, activated carbon, clay/functionalized clay, fly ash–based zeolite, aerogels, zeolites, and other adsorbents (El Alouani et al., 2021). However, researchers are continuing to seek an environmentally friendly, low-cost adsorbent with high uptake capacity that is easy to use.

Geopolymers are inorganic polymer materials derived from the alkali activation of aluminosilicate materials such as metakaolin, fly ash, and granulated blast-furnace slag (Liang et al., 2022). Geopolymers formation is often prepared through a straightforward, simple, and environmentally friendly process involving an alkali, such as NaOH/Na_2_SiO_3_ or KOH, with an aluminum and silicon-based source, thereby making it a material suitable for clean production. Geopolymers have been the subject of numerous environmental applications, partly due to their remarkable mechanical and chemical stability and their production process’ comparatively low operational energy and carbon footprint (Luhar et al., 2021). One particularly prominent use is their investigation as sorbents for wastewater treatment. Geopolymer sorbents can be installed in pipes or columns, pumping water through and interacting with the exchangeable cations on the active surface sites (Luhar & Luhar, 2021). Geopolymers have proved to be a promising category of materials for removing toxic pollutants from various source effluents. Geopolymers demonstrated high competence in providing economic and effective alternatives to conventional ceramics, synthetic zeolites, or polymeric components for wastewater treatment, in addition to being more environmentally friendly than other existing adsorbents.

In recent years, there has been an increased focus on using geopolymers as adsorbents. However, most of these review articles mainly focused on eliminating heavy metals or only one category of pollutants from contaminated water (Arokiasamy et al., 2023; Maleki et al., 2019; Rasaki et al., 2019; Tan et al., 2020; Tian et al., 2022; Tochetto et al., 2022). Additionally, these published articles outlined the geopolymer-based various precursors. Therefore, this current review article is only focuses on clay-based geopolymers, and its investigation of the performance of these geopolymers towards different existing pollutants (heavy metals, pharmaceutical compounds, dyes, and surfactants) in water. This review is also highlighting the potential of geopolymers to be an effective and low-cost solution for treating water polluted with various pollutants, making it an attractive option for reuse in industries. Thus, this review article provides an essential contribution to exploring the potential of clay-based geopolymers for circular economy initiatives.

More specifically, this review article aims to elucidate the importance of geopolymers as a modern adsorbent for removing hazardous pollutants from wastewater to obtain potable water for reuse, thereby alleviating the severe problem of water scarcity and promoting sustainable water and wastewater treatment for green ecosystems. This review provides a critical overview of the current achievements in various pollutants removal by clay-based geopolymers over the last decade. Also, this work provides an overview of a newly emerging research field and motivates further exploration of removing contaminants (heavy metals, dyes, and pharmaceutical compounds) at varied contamination levels. Moreover, the detailed physicochemical properties of various geopolymers were comprehensively reviewed and discussed. This review also covered the utilization of natural and synthetic geopolymer precursors, the mechanisms behind their removal, and the various environmental and economic benefits associated with their use. Furthermore, the role clays-based geopolymer in climate change, SDGs, environmental pollution, and the circular economy were successfully presented. Finally, the clays-based geopolymer’s existing challenges and future outlook were presented.

## Clays and clay minerals

**Fig. 2:**
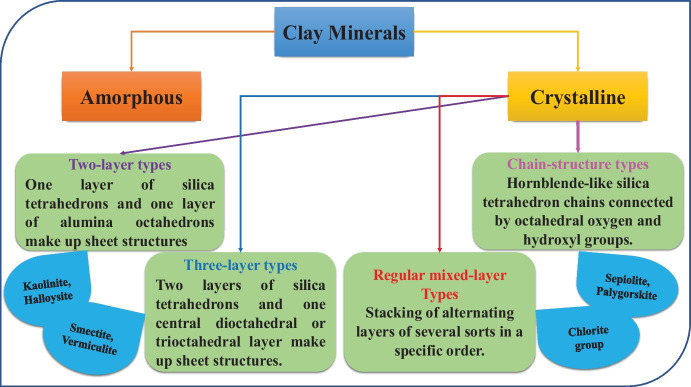
Classification of clay minerals according to Grim

Clays are hydrous aluminosilicates that combine fine-crushed clay minerals, mineral crystals, and metal oxides (Maged et al., 2023). These clays are categorized into mica, smectites, vermiculite, kaolinite, halloysite, pyrophyllite, serpentine, sepiolite, etc. (Ismael & Kharbish, 2013). Grim was the first to suggest the categorization of clay minerals, and then the delineating, terminology, and differences between several clay minerals were defined (Grim, 1962). Figure 2 demonstrates the classification of clay minerals according to Grim (Grim, 1962). According to Grim, most clays comprise one or more constituents of these three groups (Uddin, 2017).

Clays and clay minerals, in their primary forms or after modification, are known as essential materials in the present century owing to their widespread applications and many advantages, like being plentiful, low-cost, and eco-friendly resources (Wang et al., 2021b). Clay minerals are investigated by their cation exchange capacity (CEC) owing to their constant negative charge on their surfaces. Clay minerals are also classified into several types such as 2:1 type (vermiculite (Salih et al., 2022) and smectite (Gamboni et al., 2022)), 1:1 type (halloysite (Abdollahizad et al., 2022) and kaolinite (Al-Hussaini et al., 2017)), and 2:1:1 type (chlorite (Xue et al., 2021)). In the case of the 2:1 type, the negative charge is balanced by cationic counter-ions occupying interlayer space. Other cations can exchange these counter-ions, resulting in superior adsorption efficiency for cationic contaminants. These contaminants may be inorganic, such as iodine (Lazaratou et al., 2020), or organic, such as stains and pharmaceutical compounds (Jahan & Zhang, 2022). The use of clays as adsorbents demonstrated many advantages compared to the other adsorbents, such as inexpensive, availability, great surface area, robust uptake capacity, zero-toxicity nature, and the ability for ion exchange. Scientists are currently interested in using native or altered clays as adsorbents for wastewater treatment (Uddin, 2017). Recently, many studies reported different types of modified clays via the pre-treatment method to enhance the elimination capacity of some metals. This pre-treatment improves the surface area, pore size, and quantity of existing sorption locations on its external surface (Bentahar et al., 2016). The most common clay minerals used as adsorbents for various contaminants present in the aquatic environment are bentonite, montmorillonite, attapulgite, zeolites, and kaolinite. These clay minerals are characterized by promoting surface area, excellent expanding capability, and high cation exchange capacity (Otunola & Ololade, 2020).

Authors reported that bentonite clay remarkably declined the movement of heavy metals in soil by 75% (Usman et al., 2004). Other researchers confirmed that bentonite adsorption rate can reach over 99% by combining 1.0 g of bentonite with 50 mg/L of Zn(II), Cu(II), CO(II), Ni(II), and Pb(II) in 100-ml solutions (Vhahangwele & Mugera, 2015). Furthermore, zeolite is also used to treatment soils that are contaminated with Pb(II), Zn(II), and Ni(II), and the reported adsorption percentages were discovered to be in the following order Zn > Ni > Pb. Wahba et al. (2017) demonstrated that introducing a known amount of clay minerals to soil might decline plant absorption of heavy metals up to 12% (Wahba et al., 2017). Attapulgite (Palygorskite) is a 2:1 layered structure clay type with generally fibrous structures, which improves its sorption performance. Attapulgite can eliminate heavy metals from polluted medium (Samara et al., 2019). Zotiadis and Argyraki (2017) reported that the adsorption efficiency of attapulgite was found to be 100% for Cd, 21% for Ba,100% for As, 100% for Pb, 88% for Zn, and 100% for Sb in a primary schoolyard (Otunola & Ololade, 2020). For contaminated solutions, Wang et al. (2020) utilized EDTA-modified attapulgite (EDTA-ATP) for the elimination of aqueous Cr(Ш). The characterization of EDTA-ATP revealed that EDTA moieties were positively attached to the adsorbent surface. Moreover, the sorption of Cr(III) on to EDTA-ATP was found following to Langmuir model with the highest capacity record of 131.37 mg/g. XPS assessment of EDTA-ATP proved that the improved Cr(III) removal was attributed to the establishment of the stable complexes between functional (carboxyl and amino) groups of EDTA-ATP surface and Cr(III).

Sdiri et al. (2016) examined the loading capacity of montmorillonite clay for the elimination of various metal ions (Zn(II), Cu(II), Cd(II), and Pb(II)) in contaminated water (Sdiri et al., 2016). The montmorillonite efficiency as an adsorbent was confirmed using batch experiments. The authors found that the highest sorption capability was up to 131.58 mg/g, with an adsorption rate greater than 95%. Their results suggest that montmorillonite clay could be effectively used as an adsorbent for various heavy metals from water and aqueous medium (Elsherbiny et al., 2018). Malima et al. (2021) utilized the physically (thermal treatment at 300 ℃) activated Malangali kaolin clay (TAMK) to eliminate CO(II) and Cd(II) ions from artificially polluted water utilizing batch adsorption process. The used modification technique of the raw kaolinite effectively elevated its surface characteristics, such as surface area (from 78.69 to 83.05 m^2^/g) and pore volume, and amplified significantly (from 0.45 to 0.81 cc/g). The results demonstrated that the removal of CO(II) and Cd(II) is greatly affected by the variance in the experimental conditions (i.e., aging time, initial pH, adsorbent amount, and pollutant concentration in the solution). The obtained results revealed that TAMK was appropriately fitted to the Langmuir model with the highest monolayer capacity of 1.19 and 1.15 mg/L for Cd(II) and CO(II), respectively (Malima et al., 2021).

## Geopolymer and geopolymer types

In the 1970s, Prof. Joseph Davidovits launched the term “Geopolymer” to the world for the first time (Davidovits, 2020; Tan et al., 2020). The prefix ‘‘geo” represents the inorganic aluminosilicate originating from geological materials that reacted with an alkaline solution to form geopolymer from a polycondensation reaction (Liew et al., 2016). Geopolymers are mainly identified as amorphous substances with 3D alumina–silicate architecture (Açışlı et al., 2022). Geopolymers are comprised of [AlO_4_]^5−^ and [SiO_4_]^4−^ tetrahedral frameworks bounded together by sharing tetrahedral top oxygen atoms, further cations can be associated with the [AlO_4_]^5−^ groups such as Na(I), K(I), and Ca(II) ions to balance the charge (Liu et al., 2016).

**Fig. 3:**
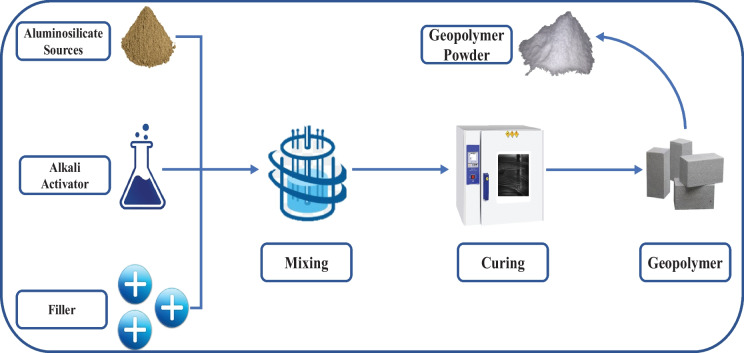
The simple synthesize process of geopolymer

Generally, geopolymer development reaction consists of non-crystalline silica and alumina-rich solids (aluminosilicate oxides) with enhanced concentrated alkaline solution to develop non-crystalline to semi-crystalline aluminosilicate inorganic substances with the 3D polymeric structure of Si − O − Al bonds, with varying Si/Al ratios (1, 2, and 3) (Verma et al., 2017). The three common types of geopolymers depend on the Si/Al ratio in the structural chains in the polymeric building units, which significantly conclude the geopolymer materials’ final configuration. When the ratio between silica and aluminum equals 1, the obtained geopolymer is called PS (− Si − O − Al − O −), while it is called PSS (− Si − O − Al − O − Si − O −) when the ratio equals 2, and PSDS type (− Si − O − Al − O − Si − O − Si − O −) is given when the ratio equal 3 (Adewuyi, 2021; Gao et al., 2014). The main principles for forming stable geopolymers are that the raw materials should be greatly amorphous, have their own reactive glassy content sufficiently, have less water demand, and release aluminum easily (Singh et al., 2015). Natural materials (i.e., kaolin, metakaolin, and zeolites) and waste products (i.e., fly ash (Verma et al., 2017), red mud (Wang et al., 2021a), rice husk ash (Barbosa et al., 2018), and blast-furnace-slag (Luukkonen et al., 2016)) can be utilized in the geopolymer preparations as the essential initial materials, manufactured via alkali or acid initiation reaction (Cong & Cheng, 2021). Figure 3 illustrates the simple synthesize process of geopolymer and geopolymerization process.

Commonly, geopolymers that can be used as adsorbents of hazardous pollutants from an aquatic environment are categorized into three sections, porous geopolymers, pervious geopolymers, and traditional geopolymers. Conventional geopolymers are un-functionalized geopolymers that include the alkalization of an aluminosilicate material such as metakaolin (El Alouani et al., 2019), treated petroleum fly ash (Al-Ghouti et al., 2021), and slag (Sarkar et al., 2017) with an activating solution such as NaOH and Na_2_SiO_3_ following by grinding process to obtain the suitable size. Porous-type of geopolymers are considered traditional geopolymers after modification by porous reagents such as H_2_O_2_ (Novais et al., 2018), surfactants (Yu et al., 2020), alginate (Yan et al., 2019a), and polyethylene glycol (Novais et al., 2019). These applied modifications resulted in a geopolymer with advanced performance and higher surface area than the conventional geopolymers. Pervious geopolymers are distinguished by their greater porosity. This form of geopolymer can be utilized in electrical, heat isolation, sound applications, solid–liquid isolation, and water treatment. This type has a low adsorption performance, less resistant in alkaline environments, and creates undesirable environmental wastes (El Alouani et al., 2021).

### Clays-based geopolymer

Clay is often chosen as the starting material to make geopolymers owing to its low-cost and accessibility in large quantities. Some noticeable research on clay-based geopolymer is focused on using kaolinite that undergoes the pre-treatment process and the resulting calcined metakaolin as initial material for synthesizing geopolymer (Tahmasebi Yamchelou et al., 2021). The chemical structure of geopolymers is well-defined as follows (Eq. 1):

1 $${\mathrm{M}}^{+} [-({\mathrm{SiO}}_{2}{)}_{\mathrm{z}}- {\mathrm{AlO}}_{2}-{]}_{\mathrm{n}}.\mathrm{ w}{\mathrm{H}}_{2}\mathrm{O}$$

where $w$ is the amount of water, $n$ is the polymerization degree, ${M}^{+}$ is the alkaline ions [Na^+^ or K^+^], and $z$ is the molar ratio of Si/Al, which can be 1–15 and even greater (Pourabbas Bilondi et al., 2018).

The geopolymer synthesis can be concluded as follows: First, aluminosilicate raw material dissolves by adding an alkali activation solution to produce silicate and aluminate monomers, and then filler can be introduced to strengthen the efficiency of the geopolymer. The appropriate initial materials must have amorphous features and the capability to release aluminum quickly. After curing at 20–100℃, the geopolymer three-dimensional network gel was achieved (Wan et al., 2017). Zhang et al. (2020) reported that increasing the curing temperature can accelerate the kinetics and geopolymerization process. The common aluminosilicate materials are fly ash and metakaolin, while alkali activators are generally a solution of KOH or NaOH and alkali metal silicate solution such as Na_2_SiO_3_ and K_2_SiO_3_, or a mixture of them (Ren et al., 2021).

The alkaline activator performs a crucial character in the generation of geopolymer. The OH^−^ ion can use as a catalyst in the reaction, and the metal cation balances the negative charge in the framework created by the tetrahedral aluminum. The smaller size and higher charge density of the Na^+^ ion compared to the K^+^ ion leads to a faster and more efficient migration through the gel network, resulting in a greater dissolution capability of NaOH than that of KOH (Lemougna et al., 2016). Consequently, these differences may change the assets of the developed geopolymers. Moreover, NaOH supports the construction of silicate monomers and dissolution of the solid aluminosilicate precursor, along with the reaction rate enhancement. In contrast, KOH can enhance the degree of polymerization (Rahier et al., 2007). Additionally, H_2_O_2_ is occasionally utilized as a foaming agent due to its thermodynamically changeable state. It readily degrades to oxygen and water, generating bubbles of gas that can be retained inside the paste and expanded to develop more homogenous holes in the matrix. It is worth noting that geopolymeric materials are formed in the same way as most zeolites are created. However, the main distinction is that once the aluminosilicate powder is combined with the alkaline liquid, a slurry is created that rapidly transforms into a hard geopolymer (Khale & Chaudhary, 2007). Therefore, unlike in the formulation of zeolites, there is not enough time or space for the gel or slurry to crystallize. Because geopolymers have a smaller setting and hardening time, the produced polycrystalline materials are closely arranged and have good mechanical strength than zeolites (Adewuyi, 2021).

Geopolymerization reaction is exothermic that can be separated into three main steps:

1. The aluminosilicate materials dissolution in the concentrated alkali solution to produce available SiO_4_ and AlO_4_ tetrahedral groups.
2. At this stage, water is released from the structure during the hydrolysis process, and then a condensation reaction of alumina and silica hydroxyl occurs to form the inorganic geopolymer gel phase **(**Eq. 2).2
3. As the gel phase hardens, AlO_4_ and SiO_4_ tetrahedral groups combine instead of develop polymorphic materials (-SiO_4_-AlO_4_-,SiO_4_-AlO_4_-, or -SiO_4_-AlO_4_-SiO_4_-SiO_4_-) by bonding all oxygen atoms among two tetrahedral units producing an amorphous to semi-crystalline geopolymer and the negative charge on Al is balanced by alkali metal cations (Eq. 3) (Cong & Cheng, 2021; Siyal et al., 2018).3

**Fig. 4:**
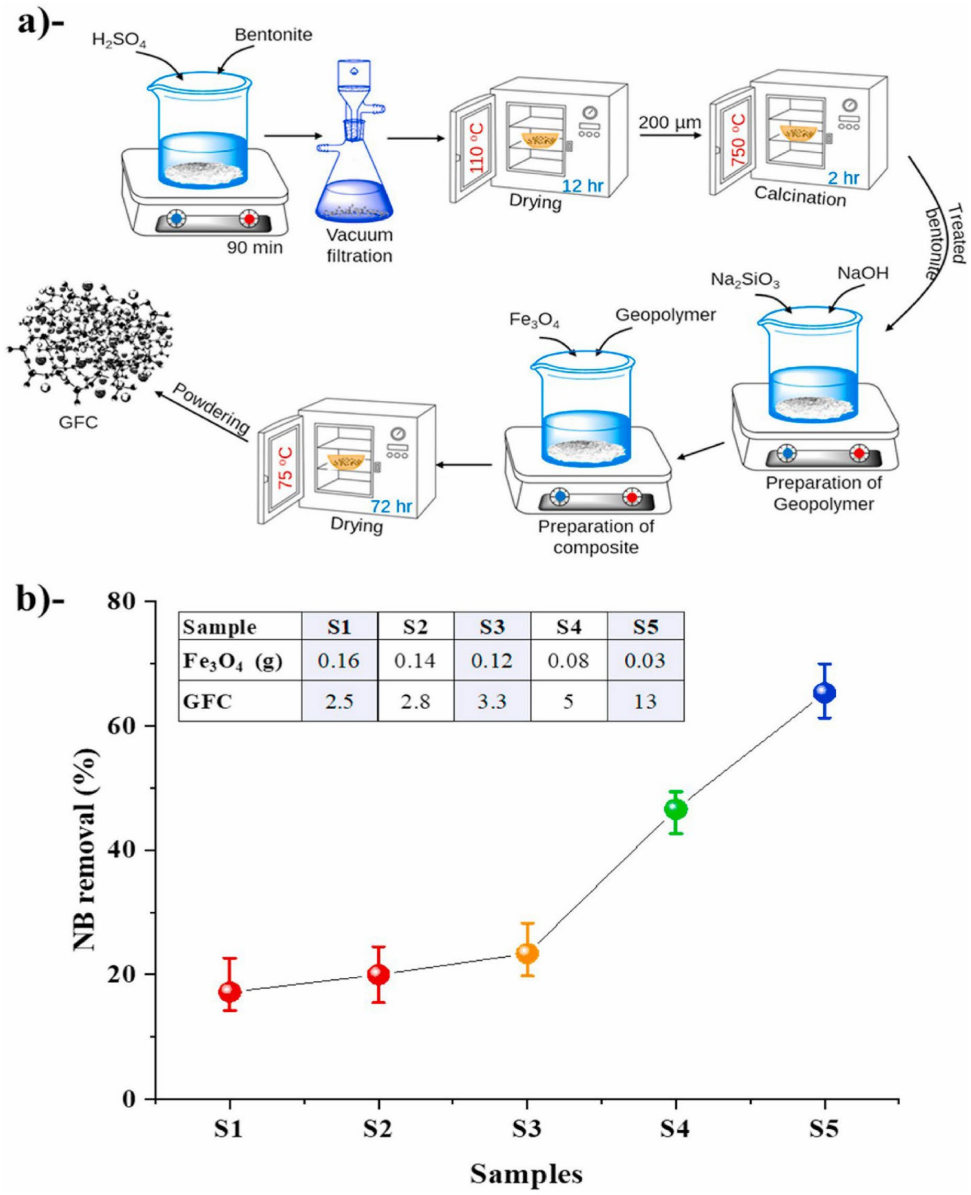
(a) Flow diagram for the synthesis of Fe_3_O_4_/geopolymer nanocomposite, **b**) effect of different magnetic nanocomposites on decolourization of Navy blue dye (NB) (%) at fixed adsorbate dosage (0.1 g), pH (5.0), and treatment time of 120 min, reused with permission from Elsevier (Khan et al., 2023)

Khan et al. (2023) investigated a bentonite clay-based geopolymer/Fe3O4 nanocomposite using advanced statistical and machine learning approaches to evaluate its adsorption capacity towards Navy blue dye. The authors performed the pre-treatment of bentonite clay to prepare the desired geopolymer. Thereafter, the synthesis of Fe_3_O_4_ nanoparticles was conducted using the co-precipitation method (Khan et al., 2023). Subsequently, the authors modified the prepared geopolymer using Fe_3_O_4_ nanoparticles via the intercalation method as follows: (i) 0.4 g of geopolymer was combined with 20 mL of deionized water and vigorously stirred until a slurry solution was formed. (ii) Different concentrations of iron magnetite nanoparticles were incorporated into the geopolymer solution, and following 30 min of constant stirring, a homogeneous mixture was produced. (iii) The nanocomposite mixture was then dried in an air oven at 75 °C for 72 h. The authors summarized the synthesis and modification process of the obtained Fe_3_O_4_/geopolymer nanocomposite in a flow diagram presented in Fig. 4.

## Geopolymers characterization

Fourier transform infrared spectroscopy (FT-IR), X-ray diffraction (XRD), Brunauer–Emmett–Teller (BET), Barrett–Joyner–Halenda (BJH), and scanning electron microscope (SEM), transmission electron microscopy (TEM), thermogravimetric (TGA), and differential thermal (DTA) analysis methods were extensively used to evaluate the functional groups, interlayer space, surface area, microstructure, structure, thermal behavior, and morphology of raw materials and geopolymers-based materials.

### XRD analysis

**Fig. 5:**
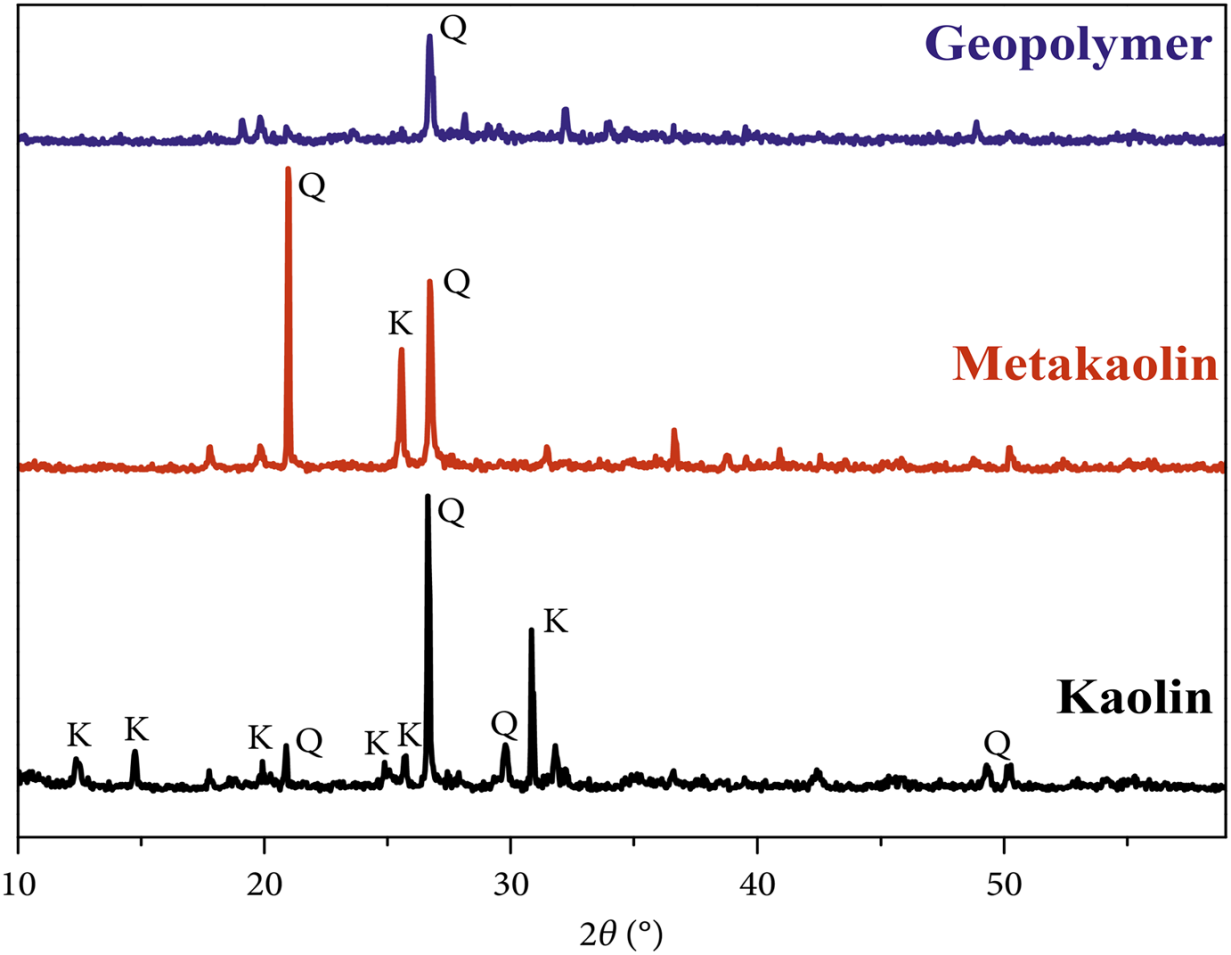
XRD patterns of kaolin, metakaolin, and metakaolin-based geopolymer (El Alouani et al., 2019)

XRD investigation was utilized to get insight into the structure of materials, whether crystalline or amorphous, prior and post-stimulation of raw materials through the geopolymerization process. Figure 5 displays the XRD patterns of kaolin, metakaolin, and metakaolin-based geopolymer. El El Alouani et al. (2019) prepared a customizable metakaolin-based geopolymer and inspected its adsorptive capacity for methylene blue removal. The XRD patterns of kaolin, metakaolin, and geopolymer demonstrated that the precursor material was dominated by quartz and kaolinite. The dehydroxylation of the water molecules can expound the absence of kaolinite characteristic peaks after the calcination process within the kaolinite structure in metakaolinite due to thermal treatment (El Alouani et al., 2019). After proceeding in the alkali activation and geopolymerization, the crystalline phases were solubilized in the basic solution, and the aluminosilicate phase was deposited onto the metakaolin surface. These findings suggested that a novel substance with a structure distinct from metakaolin had been synthesized (Fig. 5). Yu et al. (2020) performed XRD analysis for synthesized metakaolin-based mesoporous geopolymer in addition to the analysis for metakaolin and geopolymer-CTAB (Yu et al., 2020). Before geopolymerization, the ordinary MK consists of the main crystal phase, such as mullite, quartz, and kaolinite. After the geopolymerization step, the kaolinite peak decreases as the geopolymer was formed, the XRD patterns of the geopolymer and geopolymer–CTAB materials show a formless structure with a significant decrease in most of the reflection peaks (Yu et al., 2020). Khan et al. (2023) conducted XRD analysis for prepared bentonite-based magnetic geopolymer before and after Navy blue dye adsorption. The authors stated that the geopolymer showed an amorphous structure in 2θ range of 20°–37°, while the peaks at 29.2°, 35.9°, 42.9°, 57.40°, and 63.16° are characteristics of crystalline peaks related to magnetite iron oxide. The observed sharp intensities of Fe_3_O_4_ peaks were reduced in the composite due to the covering of the geopolymer (*as Fe*_*3*_*O*_*4*_* does not react during the geopolymerization*). Furthermore, the authors confirmed that no differences in the XRD pattern were observed after dye adsorption, which suggests the stability of the synthesized geopolymer for the Navy blue removal process (Rossatto et al., 2020).

### FT-IR assessment

**Fig. 6:**
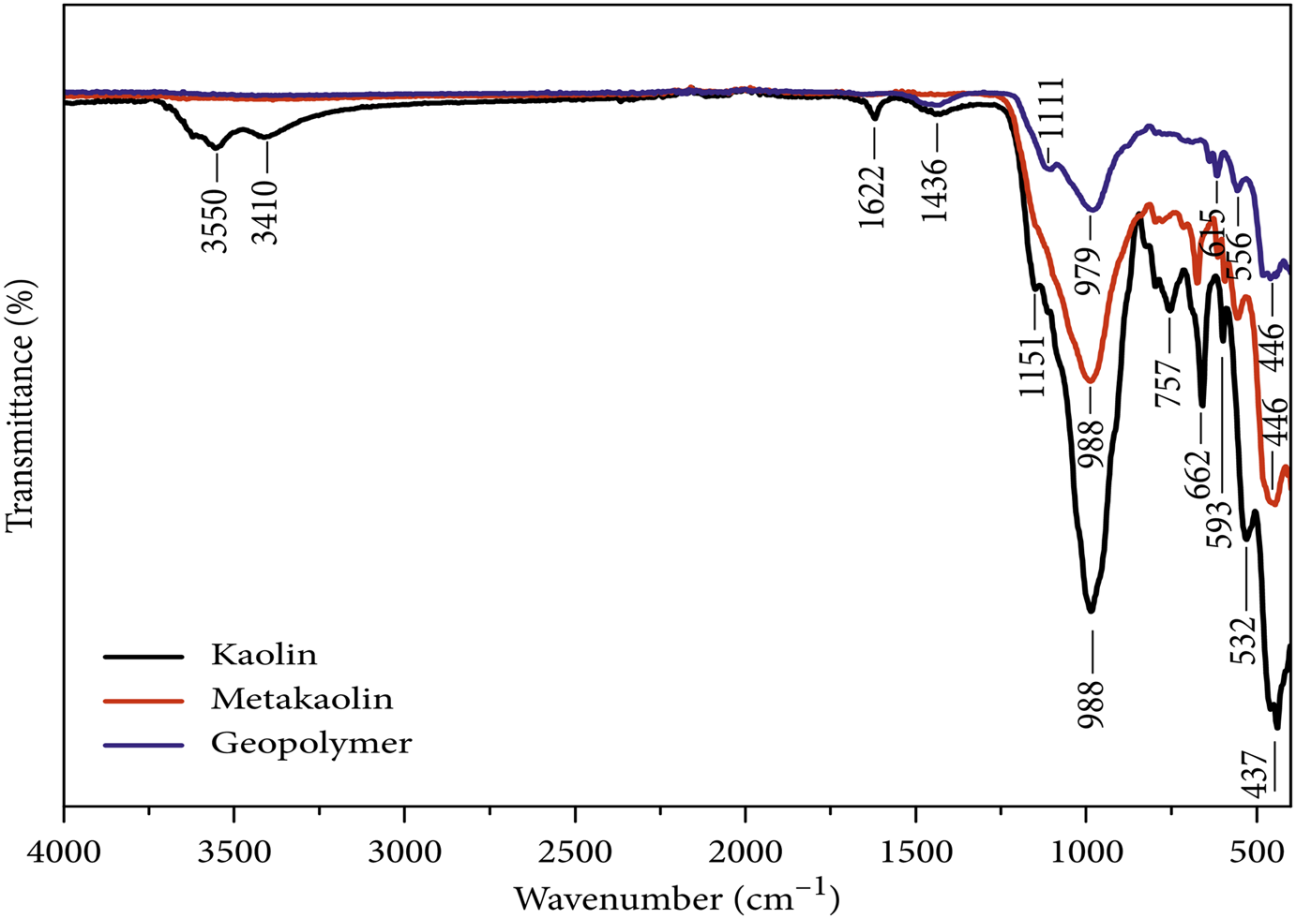
FT-IR spectrum of kaolin, metakaolin, and geopolymer (El Alouani et al., 2019)

FT-IR assessment is employed to identify the functional moieties that appear on the substance surface. Usually, most bands related to geopolymers appear at a range of 1305–910 cm^−1^ because of the asymmetric stretching modes of Si‒O‒Al. The asymmetric stretching modes of Si‒O‒Si and Si‒O‒Al show bands in the 800–400 cm^−1^ region. The peaks are located within the range of 3500–3400 cm^−1^ and 1670–1600 cm^–1^ due to stretching modes of OH and bending vibration of H‒O‒H, respectively (Rożek et al., 2018). Additionally, FT-IR spectral analysis was performed for kaolin, metakaolin, and the produced geopolymer matrix (Fig. 6) (El Alouani et al., 2019). In kaolin, the presence of weak signal bands around 1622 and 3350 cm^−1^ could be assigned to the deformation and stretching of the OH group, respectively (Maged et al., 2020b, 2023). The absorption band at 1436 cm^−1^ was designated to the O˗C˗O stretching vibrations, attributing to the atmospheric carbonation on the kaolin surface. A broadband signal at 1151 and 998 cm^−1^ intensively corresponds to the plane-stretching vibration of Si–O and stretching vibration of Si–O-Al, respectively. The additional band at 437 cm^−1^ refers to the bending vibration of the Si–O-Si group. Beyond the calcination process, no peaks were monitored in the analyzed range of 3350 and 1622 cm^−1^, which firmly proposed that the heat treatment adequately transformed the kaolin precursor into metakaolin (El Alouani et al., 2019). At the end of the geopolymerization process, the remarkable shifting (decrement) of asymmetric stretching vibration peaks spectrum characterized to Si–O-T group from 988 to 979 cm^−1^, demonstrating the success of poly(sialate-siloxo) chain assembly into the geopolymer structure (Chen et al., 2016).

Yu et al. (2020) performed FT-IR analysis for Metakaolin, geopolymer, and geopolymer-CTAB. Because of the O–H stretching and the remaining adsorbed atmospheric water, all the samples had a broad band of around 3350 cm^−1^ and a narrow band of about 1640 cm^−1^. The existence of Si–O–Si, Al–O–Si, and Si–O–Al in the octahedral structure of metakaolin is indicated by absorption peaks at 1052 cm^−1^, 714 cm^−1^, and 575 cm^−1^ in the geopolymer during the polymerization stage. The characteristics peaks at 2920 cm^−1^ and 2850 cm^−1^ in GP-CTAB reveal the existence of –CH_n_, verifying the presence of quaternary ammonium salt cations (CTA^+^) when geopolymer and geopolymer–CTAB are aligned. At the same time, the geopolymer’s characteristic peaks are still present in geopolymer–CTAB, as are the overall absorption peaks. Even if geopolymer–CTAB adds CTA^+^ during organic modification, it is proved that it still belongs to the geopolymer (Yu et al., 2020). Khan et al. (2023) performed FT-IR of synthesized geopolymer (bentonite-based magnetic geopolymer), showing that the bands at 400–650 cm^−1^ are due to Si–O–Si bending vibration and octahedral vibration Al–O–Si bending. The characteristics band at 947 and 1448 cm^−1^ demonstrates asymmetric stretching of Si–O-T (T: tetrahedral Al, Si) and the presence of carbonate, respectively. However, the bands at 1645 cm^−1^ show H–OH bending due to adsorbed water, while bands between 2900 and 3250 cm^−1^ were related to the stretching vibration of the hydroxyl group.

### Morphological assessment

**Fig. 7:**
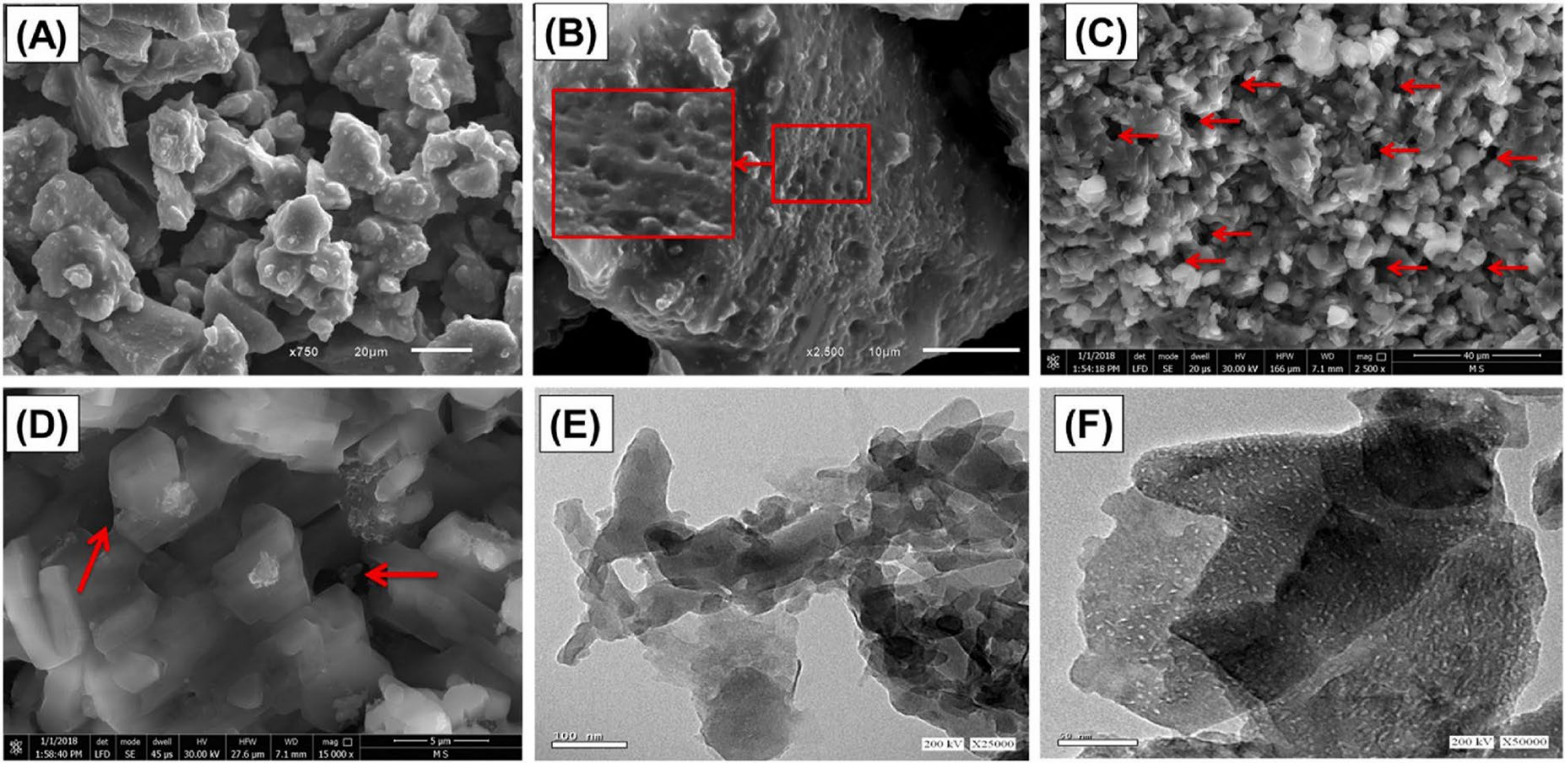
SEM image of the synthetic geopolymer (**A**), SEM image for the oriented pores in the surface of the prepared geopolymer (**B**), SEM image of the synthetic Z/GP composite (**B**), SEM image for the porous matrix of Z/GP (**D**), HRTEM image of the prepared geopolymer (**E**), and HRTEM image of the synthetic Z/GP composite with its porous structure (**F**), reused with permission from Elsevier (Salam et al., 2021)

SEM analysis offers complete details on the topographical structure of the as-employed adsorbents. Salam et al. (2021) stated that the synthesized diatomite/kaolinite geopolymer appeared in the SEM image as ceramic-like martial (*massive, irregular, and agglomerated forms without detection for cracks*) (Fig. 7A). Also, the SEM magnification proved that the synthesized geopolymer had numerous nanopores reflecting the effect of the diatomite porous frustules (Fig. 7B). Additionally, the random distribution of numerous nano-nudes on the surface of the synthesized geopolymer was a great benefit in increasing the surface area and adsorption capability. However, the zeolitized geopolymer sample affirms the presence of various crystal shapes that are typically interlocked, indicating the zeolite crystal's growth in relation to the polymeric matrix (Fig. 7C and D). The interconnected arrangement of zeolite grains within the geopolymer matrix created a highly porous surface with many interstitial nanopores, which greatly increased the surface area (Fig. 7C and D). Salam et al. (2021) prepared kaolinite/diatomite zeolitized geopolymer and systematically inspected its retention efficacy towards Sr(II) ions through batch and continuous flow systems. HRTEM investigations were carried out to assess the prepared materials’ porous structure. As shown in Fig. 7 (E), the geopolymer has a massive and aggregated monopoly, which agrees with depicting SEM images (Salam et al., 2021). Moreover, the monitored porous matrix of geopolymer may be associated with the agglomeration of its constituents grains. Otherwise, the zeolitized geopolymer appears to have a highly porous structure with multiple randomly dispersed nanopores. The synthetic zeolite’s noticed pores may have developed as considerable functional pores for the formed synthetic zeolite or the junction among its crystals during their formation inside the matrix of synthetic geopolymer (Salam et al., 2021).

Yu et al. (2020) stated that the surface of metakaolin is uneven and loose under the SEM. The dissolution of metakaolin into microparticles, caused by the activating agent’s effect, results in the formation of a geopolymer with many fine particles after the polymerization stage. Aluminum and silicon tetrahedrons be present in a free form on the metakaolin surface, where two tetrahedra contribute to one oxygen atom. The Al–O–Si structure is then rapidly polymerized into an oligomer form, followed by the silicate on the surface of the sluggish coagulation process. Figures 7B and C illustrate the geopolymer’s resultant structure. The surface of the geopolymer is regular and compact compared to metakaolin, and geopolymer–CTAB is more compact than geopolymer, possibly due to the presence of CTA^+^ (Yu et al., 2020). Moreover, Khan et al. (2023) stated that the fabricated Fe_3_O_4_/geopolymer showed a sponge-like and porous morphology. The development of the geopolymer layer has reduced the visibility of Fe_3_O_4_ nanoparticles. However, upon closer inspection, dark-bright regions indicate a core–shell morphology, such as in a composite nanosphere. This suggests that the geopolymer has effectively covered the surfaces of Fe_3_O_4_ nanoparticles (Khan et al., 2023).

### Surface area measurements

**Fig. 8:**
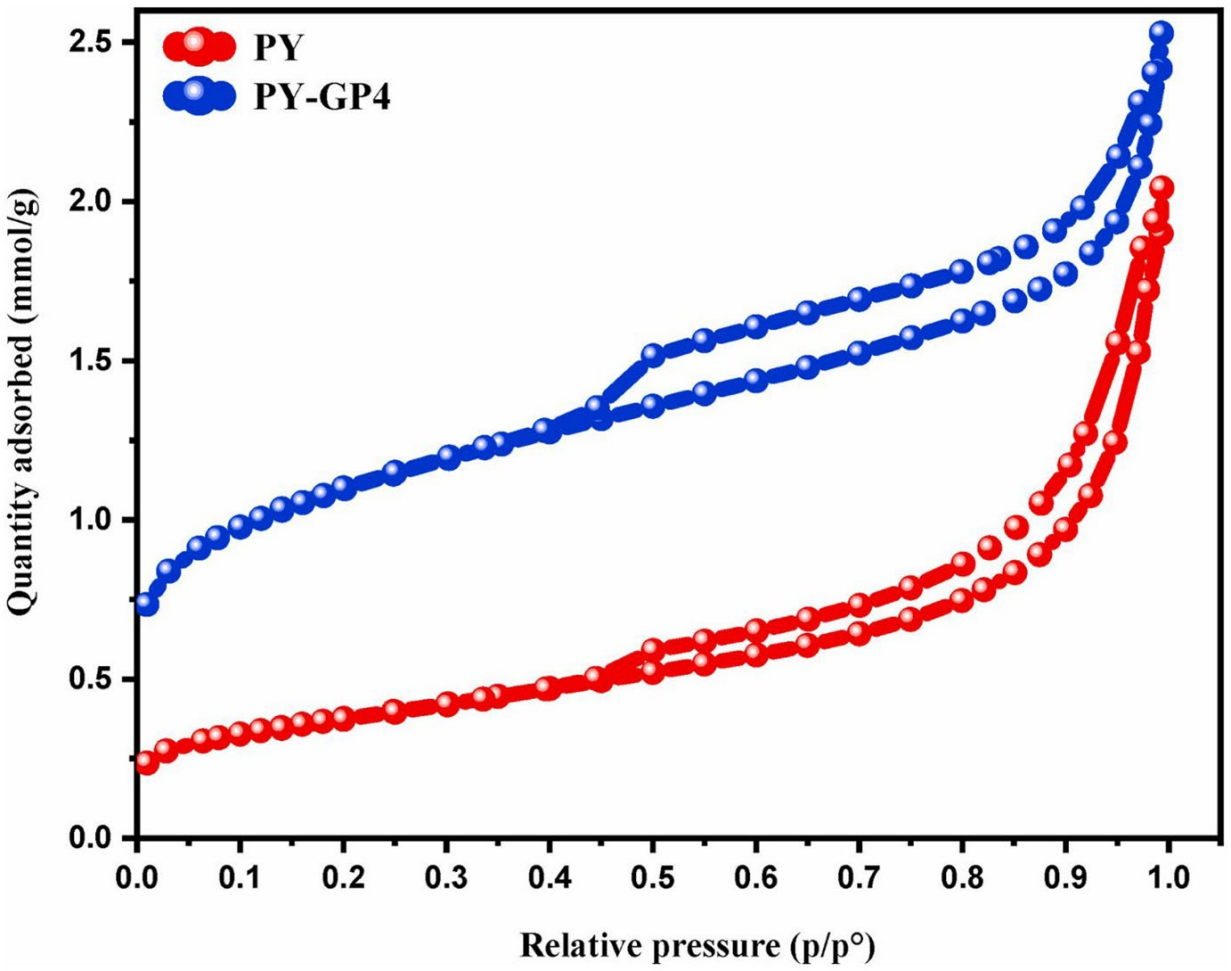
Nitrogen adsorption–desorption isotherms from the raw PY clay and the synthesized PY-GP4 geopolymer sample reused with permission from Elsevier (Ettahiri et al., 2023)

BET and BJH techniques were utilized to define the surface area, total pore volume, and average pore diameter of any materials. The BET surface characteristics of the geopolymer and MGP are tabulated in Table 2. Al-husseiny and Ebrahim (2022a) mentioned that the geopolymer’s properties were improved when the nanoparticles were precipitated. MGP increased the pore volume by 61% while increasing the initial geopolymer surface area by 62%. Furthermore, the geopolymer’s adsorptive characteristics, such as pore surface area, improved after precipitation. These results provide the first evidence that MGP could be used as an adsorbent (Al-husseiny & Ebrahim, 2022a). According to its pore structure value, MGP has a mesoporous structure with widths ranging from 2 to 50 nm. Al-husseiny and Ebrahim (2022b) have synthesized magnetite/geopolymer composite via a chemical co-precipitation strategy to remove cationic methylene blue dye. Compared to the native geopolymer sorbent, the surface properties of magnetite-loaded geopolymer composite remarkably improved. The specific surface area increased by 62.0% from 26.60 to 69.04 m^2^/g, and the pore volume (mesoporous and macroporous) enhanced by 61.0% from 6.11 to 15.86 cm^3^/g. These advancements are regarded as the first evident of the success incorporation of magnetite particles into the geopolymer structure. Moreover, the resultant composite has a mesoporous diameter ranging from 2.0 to 50.0 nm (Al-husseiny & Ebrahim, 2022b). Khan et al. (2023) evaluated the surface properties of the modified bentonite-based geopolymer composite via N_2_-sorption/desorption technique using the BET isotherm. The authors reported that the existence of type IV isotherm where the H3 Hysteresis loop indicates the aggregation of particles with plate-like structure, while the vertical asymptotic profile for high *P*/*Po* values signifies the existence of mesoporous structures with the non-uniform size and slit-shaped pores. The BET method demonstrated a surface area of 44.13 m^2^/g, an average pore size of 9.01 nm, and a total pore volume of 0.093 cm^3^/g for the modified geopolymer (Khan et al., 2023). Ettahiri et al. (2023) conducted BET measurements for pyrophyllite clay and pyrophyllite clay–geopolymer in addition to the adsorption–desorption isotherm curves as shown in Fig. 8. The authors stated that the specific surface area (*S*_BET_ = 86.5 m^2^/g) and pore volume (*V*_*p*_ = 0.08 cm3/g) for the synthesized geopolymer (PY-GP4) are both observed to be significantly greater than the values for the raw PY raw clay (*S*_BET_ = 30.0 m^2^/g, *V*_*p*_ = 0.05 cm^3^/g) (Table 2). This implies that the geopolymerization process has successfully generated an increased specific surface area and developed new micro and mesoporous sites in the final material (Ettahiri et al., 2023). Tang et al. (2015) produced low-cost, suitable, and eco-friendly porous metakaolin-based inorganic polymer spheres to remove heavy metals from aqueous solutions. The synthesis process of porous geopolymer spheres (PGS) included the optimal compositions: n(SiO_2_)/n(Na_2_O) = 1.6 in a sodium silicate solution, n(Na_2_O)/n(Al_2_O_3_) = 1, n(H_2_O)/n(Na_2_O) = 16, sodium dodecyl sulfate (K_12_) foaming agent = 1.5 mass%. Tang et al. (2015) stated that the formed geopolymer is characterized by a high surface area (53.95 m^2^/g) with a mesoporous nature (Tang et al., 2015, Yan et al., 2019a).

**Table 2:** Surface and pore characteristics and measurements of various clay-based geopolymers employed in water treatment (specific surface area (*S*_BET_), total pore volume (*V*_*T*_), and average pore width (*P*_*W*_))

Material	*S*_BET_ (m^2^ /g)	*V*_*T*_ (cm^3^/g)	*P*_*W*_ (nm)	References
Kaolin-based geopolymer	26.604	0.2372	35.66	(Al-husseiny & Ebrahim, 2022a)
Magnetite-kaolin-based geopolymer composite	69.04	0.2166	12.55	
Bentonite-based geopolymer	44.13	0.093	9.01	(Khan et al., 2023)
Cetyltrimethylammonium bromide-modified geopolymer (MGEO4)	34.900	0.138	11.69	(Açışlı et al., 2022)
Metakaolin-based geopolymer	26.4	-	30.30	(Bielecki et al., 2020)
Pyrophyllite clay	30.00	0.053	-	(Ettahiri et al., 2023)
Pyrophyllite-GP4 geopolymer	86.58	0.086	-	
Seawater-based geopolymer	11.85	0.025	-	(Padmapriya et al., 2022)
Geopolymer-NaX zeolite (G-NaX) composite	343.0	0.221	300.00 Å	(Candamano et al., 2022)
Geopolymer-NaX zeolite foamed variant (G-NaX-F) composite	752.0	0.331	500.00 Å	
Geopolymer-NaX zeolite-activated carbon (G-NaX-AC)	735.0	0.324	500.00 Å	
Geopolymer-NaX zeolite-activated carbon foamed variant (G-NaX-AC-F) composite	693.0	0.358	above 30.00 Å	
Geopolymer	1.13	0.004	14.28	(Maleki et al., 2019)
Geopolymer/Fe_3_O_4_ nanocomposite	2.32	13.76	0.01	
Kaolinite	10.0	0.072	43.20	(Salam et al., 2021)
Diatomite	117.7	0.032	5.14	
Diatomite/kaolinite-based geopolymer composite	89.4	0.039	11.60	
Diatomite/kaolinite-based zeolite/geopolymer composite	106.0	0.058	4.20	
Pure geopolymer (KGP)	17.59	0.201	45.18	(Yan et al., 2019b)
Hollow gangue microspheres (GM)	6.03	0.043	28.39	
GM/KGP	26.41	0.330	49.37	
MK	3.11	0.015	1.56	(Yu et al., 2020)
GP	31.83	0.078	3.22	
GP-CTAB	26.45	0.121	9.12

### TGA/DTA analysis

TGA/DTA analysis is an essential technique for understanding the properties of clay-based geopolymer adsorbents. It is used to characterize the thermal properties of the synthesized geopolymer by measuring the temperature and weight changes during heating, which can reveal important information about the structural and thermal properties of the adsorbent. Moreover, TGA/DTA data can be used to determine the optimal conditions for synthesizing and activating the adsorbent and optimize the adsorption performance. Therefore, TGA/DTA analysis is essential for designing and optimizing clay-based geopolymer adsorbents.

**Fig. 9:**
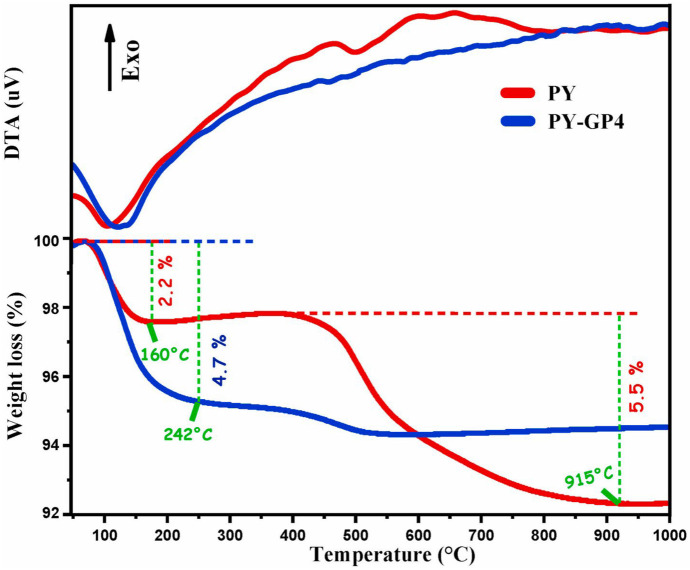
TGA/DTA curves of PY raw sample and PY-GP4 prepared geopolymer reused with permission from Elsevier (Ettahiri et al., 2023)

Ettahiri et al. (2023) performed the TGA/DTA analysis data for the raw pyrophyllite (PY) clay and the synthesized pyrophyllite clay-based geopolymer (PY-GP4) as a function of the heating temperature. The PY data indicates the occurrence of two endothermic transitions (Fig. 9). The first, detected between 50–110 °C, is connected to a 2.2% mass loss (Maged et al., 2023). This can be attributed to the expulsion of water that had been adsorbed to the particle surfaces and pores of the clay minerals. The second endothermic transition, observed between 420 and 915 °C (Fig. 9), is associated with a 5.5% mass loss and is attributed to the dehydroxylation of the pyrophyllite, kaolinite and muscovite clays minerals found in the raw sample (Erdemoğlu et al., 2020). The authors reported that the mass loss of physisorbed water in the PY-GP4 geopolymer was found to be 4.7%, which was higher than that of the raw PY sample, suggesting that the geopolymer synthesis conditions led to the development of porosity and permeability (Fig. 9). The mass loss observed above 400 °C for the PY-GP4 geopolymer (0.4%) suggests a high degree of stability due to the utilization of PY-800 (metapyrophyllite) as a precursor phase for the geopolymer formation (Ettahiri et al., 2023). However, Khan et al. (2023) conducted TGA analysis for the bentonite clay and Fe_3_O_4_/geopolymer nanocomposites (GFC) in terms of weight loss (%) at constant heating rate of 10 °C/min (up to 1000℃). The TGA analysis reveals that GFC exhibited two considerable mass decrease stages compared to bentonite clay. The authors stated that the initial 10% weight loss from 50 to 125 °C in the sample evidenced a removal of moisture and impurities, while the subsequent 28% loss between 130 and 850 °C is attributed to the decomposition of carbonates in GFC (2^nd^ stage), resulting in a total weight loss of approximately 32% (Hajizadeh et al., 2020). Khan et al. (2023) reported that the weight loss analysis of GFC compared to bentonite clay in the temperature range of 50–125 °C demonstrated that the polymeric structure of GFC has considerable absorbency for water and impurities. Nonetheless, a negligible weight decrease of the GFC was observed from 850 to 1000 °C, indicating that pyrolysis took place in the examined temperature range (Khan et al., 2023). Moreover, Ghani et al. (2020) fabricated a novel geopolymer form activated laterite clay for the Ni(II) and Co(II) ions from aqueous solutions.

## Application of clays and geopolymers in water treatments

### Heavy metals removal

Cheng et al. (2012) reported that a metakaolin-based geopolymer formed from calcination of kaolinite clay at 650℃ mixed with an alkaline solution of NaOH and sodium silicate with a molar ratio of SiO_2_/Na_2_O equivalent to 1.0. The authors examined the uptake efficiency of the geopolymer for numerous metals such as Cu(II), Pb(II), Cd(II), and Cr(III) in aqueous solutions under various experimental parameters. From the examined these metal ions, the results confirmed that the synthesized geopolymer could adsorb the targeted ions, and the best adsorption capacity (147.06 mg/g) was investigated for Pb(II) (Cheng et al., 2012). Chen et al. (2013) fabricated zeolite-enhanced geopolymer material by using metakaolin and fly ash as raw materials for the elimination of Cs(I), Co(II), and Sr(II) from water by a static method. The uptake capacity of the developed geopolymer was affected by the solution’s ion concentration and pH value. Cs(I), Co(II), and Sr(II) desorption could be stimulated by immersing the adsorbents in low pH or high ionic strength solutions. The adsorption process of Cs(I), Co(II), and Sr(II) ions onto the obtained geopolymer was proved to be following the first-order reaction. While the adsorption isotherm curves indicated that the Langmuir model was the best-fitted model for describing the process with maximum uptake capacity of 564.97, 152.91, 154.32 mg/g for Cs(I), Co(II), and Sr(II), respectively (Chen et al., 2013).

Luukkonen et al. (2016) studied metakaolin and blast-furnace-slag geopolymers along with their precursors for the elimination of Sb(III), As(III), and Ni(II) from spiked mine discharge. Based on the obtained adsorption results, Luukkonen et al. (2016) found that the blast-furnace-slag geopolymer is the highest uptake efficiency among the studied adsorbent materials with 3.74, 0.52, and 0.34 mg/g, for Ni(II), As(III), and Sb(III), respectively. The authors stated that although the adsorption capacities were comparatively modest owing to the complicated water matrix, a high elimination percentage (90–100%) of Sb(III), Ni(II), and As(III) was reached with increasing the geopolymer dosage appropriately. Luukkonen et al. (2016) concluded that geopolymer could turn blast-furnace slag into an efficient adsorbent material with a particular application in the mining industry (Luukkonen et al., 2016). Novais et al. (2016) reported a synthesis process of a novel porous biomass fly ash–containing geopolymer monoliths using a mixture of metakaolin and biomass fly ash to determine the feasibility of employing these materials for the elimination of Pb(II) ions under various conditions. The experiments showed that the adsorption capacity of Pb(II) ions was affected by the porosity of the geopolymer and the pH of the working solution. The maximum Pb(II) adsorption capacity extended between 0.95 and 6.34 mg_lead_/g_geopolymer_. These innovative geopolymeric monoliths could be employed easily in packed beds (continuous flow mode), which can conveniently collect the spent adsorbent. This advantage significantly benefits the synthesized geopolymer monoliths over powdered adsorbents (Novais et al., 2016). Furthermore, the fabrication process of this monolith incorporates the reuse of biomass fly ash, reducing the environmental impact of waste disposal and lowering adsorbent production costs (Novais et al., 2016).

Moreover, a novel geopolymer/alginate hybrid spheres (GAS) was formed from green fabricated metakaolin-based geopolymer combined with sodium alginate using an appropriate one-pot method to introduce negatively charged carboxyl groups (Ge et al., 2017). The authors assumed that the diffusion of positively charged metallic ions inside the adsorbent was enhanced by the negatively charged carboxyl groups immobilized onto the polymeric matrix through electrostatic attraction. The fabricated material (GAS-4) was used as an adsorbent to uptake Cu(II) from an aqueous solution with 62.5 mg/g. The authors also applied geopolymer/alginate hybrid spheres in a dynamic mode for the continuous flow treatment of Cu(II) effluents. Ge et al. (2017) stated that the GAS-4 would be eco-friendly and economical and could remediate heavy metals in water on a continuous flow system (Ge et al., 2017). Metakaolin-based geopolymer (MKG) was fabricated by dissolving Mefisto L05 metakaolin in an alkaline solution of NaOH and sodium silicate with molar ratios of Na_2_O/SiO_2_ = 0.2, SiO_2_/Al_2_O_3_ = 3.2, Na_2_O/Al_2_O_3_ = 0.7, and H_2_O/Na_2_O = 13.8 (Kara et al., 2017). The synthesized geopolymer was fabricated to remove Zn(II) and Ni(II) ions from an aqueous solution. The authors stated that the adsorption system investigated the various parameters (i.e., initial pH, the quantity of adsorbent, contact time, and initial cation concentration on the metal removal efficiency of MKG) using a batch method at 25 °C. Kara et al., (2017) concluded that the adsorption capacity increases with increasing contact time while the capacity decreases with increasing MKG amount. The obtained adsorption data were found following the Langmuir isotherm model with estimated maximum monolayer capacities of 7.26 × 10^−3^ and 1.14 × 10^−4^ mol/g for Ni(II) and Zn(II), respectively. Kara et al., (2017) also conducted continuous flow adsorption mode and demonstrated that the optimal flow rates for Ni(II) and Zn(II) were 1.0 and 2.0 mL/min, respectively. However, the breakthrough curve points were detected at 4.67 and 2.50 h for Ni(II) and Zn(II), respectively. Kara et al. (2018) proposed removing manganese (Mn(II)) and cobalt (Co(II)) ions from aqueous solutions by fabricating a low-cost metakaolin-based geopolymer with activating agent composed of a mixture of sodium hydroxide and sodium silicates. The fabricated geopolymer demonstrated maximum adsorption capacities of 1.32 × 10^−3^ mol/g and 1.18 × 10^−3^ mol/g for Mn(II) and Co(II) ions, respectively. The adsorption efficiency of the obtained geopolymer showed slight sensitivity toward temperature (below 30 °C) and ionic strength (> 0.04 mol/g), while pH was unnecessary to be adjusted (Kara et al., 2018).

On the other hand, green and inexpensive geopolymer (GM/KGP) with hollow gangue microspheres present in the matrix produced for the elimination of heavy metal ions such as Cu(II), Cd(II), Zn(II), and Pb(II) from wastewater (Yan et al., 2019b). The effect of contact time, temperature, and geopolymer dose was investigated to evaluate the removal capacity. The authors mentioned that GM/KGP demonstrated a characteristic broad amorphous structure with a typical rich functional (O-containing) group on the geopolymer surface. Yan et al. stated that various adsorption mechanisms were ascribed for the adsorption process onto GM/KGP, including ion exchange, physical, chemical, and electrostatic attractions (Yan et al., 2019b). The microspheres were regularly dispersed in the geopolymer matrix, leading to an increased BET surface area value of 26.41 m^2^/g. The results show the adsorption capacity of heavy metal ions was in the following order: Pb(II) > Cu(II) > Cd(II) > Zn(II) → 138.89, 18.25, 17.58, 14.31 mg/g, respectively (Yan et al., 2019b). Maleki et al., (2019) utilized magnetic nanocomposite-based geopolymer for the removal of heavy metals such as Cu(II), Pb(II), Ni(II), Cd(II), and Hg(II) from industrial wastewaters. The authors used bentonite clay in the manufacturing process as it is naturally available, has a low-cost, high surface area, Al_2_O_3_/SiO_2_ ratio, amorphous content, and is a chemically stable material. The synthesized geopolymer was modified by adding Fe_3_O_4_ nanoparticles. After that, the geopolymer/Fe_3_O_4_ nanocomposite was used to efficiently remove the targeted heavy metals from an aqueous solution. The obtained geopolymer exhibited 99%, 99%, 92%, 96%, and 92% elimination efficiency for Cu(II), Pb(II), Ni(II), Cd(II), and Hg(II) from industrial wastewaters, respectively. Maleki et al. (2019) reported that the superior adsorption capacity was achieved in 2 min using 50 mg of the geopolymer nanocomposite.

Yu et al. (2020) utilized cetyltrimethylammonium bromide (CTAB) in a unique and easy synthetic pathway as an organic modifier for the production of metakaolin-based mesoporous geopolymer (GP-CTAB) for the simultaneous removal of Cu(II) and Cr(VI) from water. The results indicated that GP-CTAB could adsorb anions at the same time without losing the adsorption properties of heavy metal cations. The higher adsorption capacity of GP-CTAB for Cr(VI) and Cu(II) was 95.3 and 108.2 mg/g, respectively. Interestingly, the authors mentioned that the presence of Cu(II) ions in the solution enhanced the adsorption of Cr(VI) (Yu et al., 2020). Lan et al. (2020) reported detailed steps for the synthesis of geopolymer with a specific surface area of 82.8 mg/m^2^ by mixing coal fly ash as raw material (35 wt.%) and metakaolin (65 wt.%) for the uptake of Pb(II) and Cd(II) ions from an aquatic environment. The results confirmed that the formed geopolymer has a maximum removal capacity of 164.10 and 78.20 mg/g for Pb(II) and Cd(II), respectively. The authors also mentioned that the obtained geopolymer generates high-performance adsorbent material and is a productive technique for using industrial waste resources (Lan et al., 2020).

Tan et al. (2021) reported another porous geopolymer sphere produced to remove Ni(II) from wastewater using fly ash and calcined kaolin as raw materials. Tan et al. (2021) concluded that the Ni(II) adsorption capacity increases 3.4 times in the case of porous geopolymer spheres with contact time of 48 h and pH value of five compared to the non-foamed geopolymer. The enhancement in the adsorption capacity (19.94 mg/g) refers to the large pore size that allows easy diffusion of Ni(II). The authors confirmed that the obtained geopolymer sphere could be employed directly in fixed-bed (continuous flow) systems without any supporting medium (Tan et al., 2021).

The removal of Ni(II) ions and methylene blue by metakaolin-based geopolymer was reported by Jin et al. (2021). The metakaolin-based geopolymer exhibited more negative charge adsorption sites after the pH adjustment, causing a quick removal of MB and Ni(II) ions. Desorption of Ni(II) ions was promoted by decreasing the pH of the wash solution. Nevertheless, MB-loading geopolymer proved to be difficult for the desorption process. The authors gave a global reference and the prospect of quickly removing cationic dyes and heavy metal ions under relatively mild conditions (Jin et al., 2021).

Diatomite/kaolinite-based geopolymer was produced and integrated into the zeolitization method by Salam et al. (2021). The authors examined the role of the zeolite materials in the adsorption of Sr(II) ions dissolved in water. Batch and fixed-bed column studies were used to compare the adsorption of Sr(II) ions using zeolite-based and original geopolymers. The results implied that the adsorption capacity of zeolite-based geopolymer in the batch study was 193.7 mg/g, higher than the normal geopolymer (102 mg/g). Also, Salam et al. (2021) studied the possibility of recycling the formed geopolymers after usage. They found that zeolite-based geopolymers had more excellent stability than normal geopolymers, with an adsorption efficiency of over 90% for five use cycles. The adsorption reaction of Sr(II) ions is spontaneous and exothermic, stimulating the reaction to progress at a low temperature of 20 °C. The column studies also showed higher adsorption in the case of zeolite-based geopolymer (72.9%) with a saturation time of 27 h for treating a contaminated solution of 8 l (Salam et al., 2021).

### Dyes removal

A study on the removal of methylene blue from wastewater was explored by Khan et al. (2015). The authors recommended the application of phosphoric acid in the synthesis of metakaolin-based geopolymers. Two different phosphoric acid–based geopolymers were synthesized with molar ratios (phosphoric acid: alumina) of 1:1 and 1.2:1. The highest sorption capacities of geopolymer-1 M and geopolymer-2 M were recorded to be 2.84 and 3.01 mg/g, respectively. Phosphoric acid–based geopolymers could be restored by furnace treatment at 400 °C for 2 h, increasing uptake capacities up to 5.07 mg/g for five repeated cycles (Khan et al., 2015).

Zhang et al. (2016) investigated the use of 2-D graphene (GR) and blast-furnace-slag-based geopolymer (ASG) initiated by alkaline solution for the synthesis of novel graphene/geopolymer nanocomposite (GR/ASG) (Zhang et al., 2016). The GR/ASG1 sample’s pore diameter distribution revealed that 89.59% of the pore volume was centered in the 2–50 nm range. Under UV irradiation, the photocatalytic degradation efficiencies of sorbents for methyl violet dye are in the order GR/ASG1 (91.16%) > GR/ASG2 (86.12%) > ASG (80.48%). The maximum degrading efficiency (91.16%) was reached after 110 min by GR/ASG1 photocatalyst (Zhang et al., 2016).

Furthermore, the removal of methyl violet 10B dye from aqueous solutions was successfully achieved by using mesoporous geopolymer synthesized from a mixture of metakaolin and rice husk ash as aluminosilicate source and soybean oil as a mesostructured-directing agent (Barbosa et al., 2018). The experiment was conducted with a geopolymer sample with and without soybean oil. The mesoporous geopolymer was extensively characterized. The results displayed that the presence of oil is effective and vital, as the adsorption efficiency shows excellent performance with the existence of oil in the fabricated geopolymer due to its greater pore properties. The maximum adsorption capacity of mesoporous geopolymer was reported to be 276.90 mg/g (Barbosa et al., 2018). El Alouani et al. (2019) studied the capability of using geopolymer in the powder form produced by using metakaolin as raw material activated by Na_2_SiO_3_ powder and NaOH to remove the cationic dye, particularly methylene blue from water. The experimental data showed that geopolymer could effectively remove methylene blue at a high pH value, and the maximum adsorption was found to be 43.48 mg/g. the adsorption process occurs spontaneously as an endothermic process (El Alouani et al., 2019).

Hua et al. (2020) studied using magnetic geopolymer to remove dyes that polluted water, especially procion red and acid green. The investigational data implied that the sorption of acid green (186.18 mg/g) was higher than procion red (40.35 mg/g). The authors also studied the effect of temperature on the uptake capacity of geopolymer. They found that the temperature had little impact on the difference in adsorption capacity for both dyes. Hua et al. (2020) stated that the adsorption energies implied that physical interactions were involved during the sorption mechanism of the targeted dye pollutants (Hua et al., 2020). Rossatto et al. (2020) synthesized a novel magnetic geopolymer/Fe_3_O_4_ composite using H_2_O_2_ and soybean oil to create a geopolymer with a mesoporous structure from metakaolin, biogenic rice husk silica, and Fe_3_O_4_. To maximize the adsorption effectiveness of the acid green 16 dye onto magnetic geopolymer, the experimental design was used to analyze the parameters such as geopolymer dose and initial pH. The data demonstrated that rapid adsorption and equilibrium were attained after 30 min. At 328 K, a remarkable adsorption capacity of 400 mg/g was achieved. Moreover, despite numerous repeated cycles, the composite showed high reusability, indicating that it could be applied as a sorbent to remove organic contaminants from liquid effluents (Rossatto et al., 2020). Pimraksa et al. (2020) described the process of geopolymers fabricated from metakaolin as an aluminosilicate source with a molar ratio of 2.5 related to SiO_2_/Al_2_O_3_ in forming a geopolymer–zeolite composite. Different quantities of zeolite and TiO_2_ were trapped in the geopolymer matrix. Pimraksa et al. (2020) stated that the results showed that the pulverized geopolymer composites with 40 wt.% TiO_2_-doped zeolite had better adsorption capacity with 99.1% than geopolymer with 40 wt.% without doped TiO_2_ in their frameworks achieved 92.5% as the maximum uptake capacity for methylene blue removal (Pimraksa et al., 2020). Furthermore, the continuous usage of geopolymer with TiO_2_-doped zeolite as photocatalysts revealed superior stability. On the other hand, the pelletized geopolymer composites had a low surface area which in turn reduces the efficiency of adsorption of methylene blue.

Moreover, Al-husseiny and Ebrahim (2022b) reported using metakaolin-based geopolymer to form magnetite/geopolymer composite via a chemical co-precipitation technique to remove methylene blue from water. To improve the removal efficiency of geopolymer, magnetite was precipitated on the surface of the geopolymer. The surface area, chemical resistance, pH, time of contact, and initial concentration of methylene blue were all significant parameters in methylene blue removal from wastewaters by magnetite/geopolymer composite. The geopolymer and Fe_3_O_4_/geopolymer composite surface areas were 26.604 and 69.04 m^2^/g, respectively. The magnetite/geopolymer composite showed much better adsorption efficiency than mesoporous geopolymers. A higher removal efficiency of over 95% was achieved when the geopolymer was mixed with 10% Fe_3_O_4_ (Al-husseiny & Ebrahim, 2022b).

### Organic compounds removal

#### Pharmaceutical compounds

Only a few studies have been published using geopolymers and modified geomaterials to remove pharmaceutical compounds from aquatic environments. Recently, magnetite/geopolymer composite was used as a new adsorbent of pharmaceutical compounds, especially antibiotic drugs (tetracycline) from wastewater. Al-husseiny and Ebrahim (2022a) produced magnetite/geopolymer composite using a chemical co-precipitation technique. The surface area of geopolymer and magnetite/geopolymer composite was reported to be 26.60 and 69.04 m^2^/g, respectively. The authors studied the role of Fe_3_O_4_ after precipitating on the geopolymer surface. The authors found that magnetite/geopolymer composite with 10% Fe_3_O_4_ had superior adsorption performance towards tetracycline, and the removal efficiency of the fabricated geopolymer could reach over 90%, which was substantially higher than that of individual Fe_3_O_4_ and geopolymer (Al-husseiny & Ebrahim, 2022a). Furthermore, Wang et al. (2022) innovated a modified metakaolin-based geopolymer microsphere using oleic acid aiming for a superior adsorption performance for Tetracycline pharmaceutical compound from saline water. The authors stated that the oleic acid modification technique effectively enhanced the fabricated modified geopolymer’s surface area and pore volume. Moreover, the obtained geopolymer exhibited a maximum Langmuir uptake capacity of 645.70 mg/g at 298 K. Additionally, the fabricated geopolymer showed an excellent uptake performance in the saline medium and demonstrated the applicability to be regenerated. Also, the innovative geopolymer was successfully utilized in a continuous flow adsorption system. Wang et al. (2022) mentioned that ion exchange, electrostatic interactions, and hydrogen–bonding interactions were involved through the adsorption mechanism.

#### Surfactants

Recently, the existence of surfactants in global water resources has increased dramatically. Consequently, designing an appropriate adsorbent, such as geopolymers, to eliminate these anionic surfactants from manufacturing and domestic effluents before their release into freshwater resources was of great interest, considering the concentration limits and high consumption rate of these pollutants. Clay-based geopolymers have not been identified in the literature to treat surfactants from aqueous solutions; however, other geopolymers have been demonstrated to remove these surfactants from water effectively.

Siyal et al. (2019) reported an economical and straightforward process for fabricating fly ash–based geopolymer targeting the removal of sodium dodecyl benzene sulfonate (anionic surfactant) from an aqueous medium using a batch system. The morphological analysis showed that the fabricated geopolymer possessed a surface area of 31.87 m^2^/g. Moreover, an adsorbent dosage of 1 g/L, contact time of 180 min, and solution pH ≈ 2 was enough to achieve the highest uptake capacity of 714.3 mg/g. Siyal et al. (2019) suggested that the adsorption process of sodium dodecyl benzene sulfonate was chemisorption and physisorption along with monolayer adsorbable coverage based on the kinetics, thermodynamics, and isotherms studies. Additionally, electrostatic interactions were the involved mechanism for sodium dodecyl benzene sulfonate onto the obtained fly ash–based geopolymer. Siyal et al. (2021) prepared a porous fly ash–based geopolymer to remove an anionic surfactant (sodium dodecylbenzene sulfonate). The authors used the response surface methodology by changing the synthesis parameters of Si/Al, Na/Al, and water-to-solid (W/S) ratios and the removal efficiency as the obtained response. The microstructural analysis verified the existence of rods, pores, and cavities in the fabricated geopolymers with a surface area of 59.51 m^2^/g. Siyal et al. (2021) stated that the synthesized geopolymer exhibited high uptake capacity of 743.71 mg/g with total elimination efficiency of 84.50% towards sodium dodecylbenzene sulfonate. Strozi Cilla et al. (2014) confirmed that the presence of anionic surfactant on the geopolymer significantly improves geopolymer chemistry and consequently enhances its surface area.

Clay-based geopolymers have been demonstrated to have a superior capacity for removing heavy metals, dyes, and pharmaceutical compounds from aqueous solutions. This is due to the high surface areas and adsorption capacities of geopolymer materials, resulting from their nanostructure and active function group, which provide a high affinity for the adsorption of such contaminants. Furthermore, the ability of clay-based geopolymers to undergo a pH-dependent structural transformation, which increases their adsorption capacity, makes them particularly suitable for removing such compounds from aqueous solutions. In the case of heavy metals, laterite clay–based geopolymer showed the highest adsorption capacity (520.00 mg/g) compared to the other clay-based geopolymers found in the literature (Ghani et al., 2020). Also, the zeolite-enhanced geopolymer material demonstrated a maximum uptake capacity of 564.97 mg/g for Cs(I) removal from an aqueous solution (Chen et al., 2013). Moreover, the organically modified metakaolin-based mesoporous geopolymer (GP-CTAB) revealed a high sorption capacity (164.10 mg/g) for Pb(II) ions (Lan et al., 2020). In the case of dye removal, the metakaolin-based geopolymer (magnetic/zero-valent iron) showed a superior sorption capacity of 1814.0 mg/g towards Acid Red 97 dye (Netto et al., 2020). Also, the metakaolin-based geopolymer (mixed with rice husk ash) has a good sorption capacity of 276.9 mg/g for Methyl violet 10B dye (Barbosa et al., 2018). Regarding pharmaceutical compound removal, only a few studies were found in the literature. However, the metakaolin-based geopolymer microspheres (oleic acid modified) and kaolinite-based geopolymer showed a good adsorption capacity (645.7 and 329.7 mg/g) towards tetracycline (Wang et al., 2022) and 5-fluorouracil drugs (Abukhadra et al., 2022), respectively.

### Adsorption mechanism of clay-based geopolymers

Adsorption is essential in many industrial processes, and understanding and controlling any adsorbent’s adsorption mechanism is vital to optimizing these processes. Adsorption is a physical and/or chemical process in which molecules of a substance, such as a gas, liquid, or solid, are attracted to the surface of adsorbent material. Understanding this mechanism is vital to designing effective adsorbent materials, characterizing adsorption processes, and developing adsorption-based separations and purifications. The adsorption mechanism determines the capacity of an adsorbent for a given substance, its selectivity for different substances, and the rate at which adsorption occurs. It also affects the equilibrium adsorption of a substance as a function of the adsorbate’s temperature, pressure, and composition. An improved understanding of the adsorption mechanism of any adsorbent will allow for the design of more efficient adsorbents for a wide range of applications, such as water purification, environmental remediation, and separations and purifications in the food, pharmaceutical, and biotechnological industries.

**Fig. 10:**
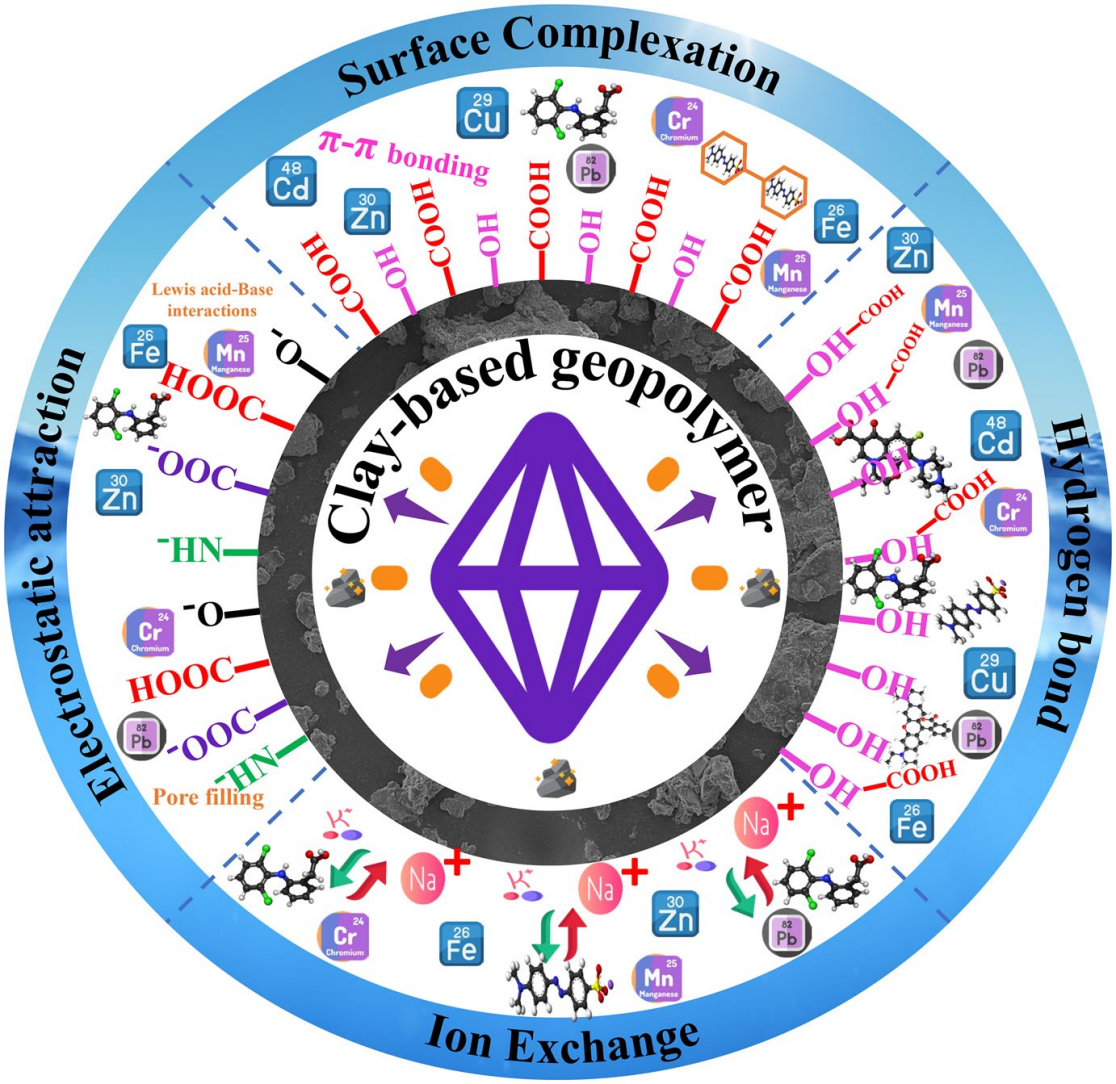
Possible adsorption mechanisms of clay-based geopolymers for heavy metals, pharmaceutical compounds, and dyes

Clay-based geopolymers are attractive materials for use as adsorbents due to their large surface area, porosity, and chemical stability. The adsorption mechanism of clay-based geopolymer materials is affected by several factors, including the surface chemistry, pore size and structure, and the interactions between the adsorbent and adsorbate. The surface of geopolymer materials can be either hydrophilic or hydrophobic, and the nature of the surface will affect the type of adsorption mechanism that occurs (Tan et al., 2020). Based on the type of bonds formed between the adsorbate and adsorbent, two major adsorption mechanisms can be identified: physisorption and chemisorption. Physisorption involves physical forces such as van der Waals forces, while chemisorption is characterized by the formation of chemical bonds. Additionally, other adsorption mechanisms, such as ion exchange, ion pairing, electrostatic attraction, hydrophobic bonding, hydrogen bonding, and dispersion forces, may also occur at solid–liquid surfaces (Siyal et al., 2018). It is believed that a combination of physical and chemical interactions, including hydrogen bonding and electrostatic forces, is responsible for the adsorption of molecules to the surface of geopolymer materials. The presence of functional groups, such as carboxylate, hydroxyl, and amine groups can further enhance the adsorption of molecules to the surface of geopolymer materials. These functional groups can increase the affinity of the adsorbent for polar molecules and the adsorption rate. Figure 10 illustrates the possible adsorption mechanisms of clay-based geopolymers for heavy metals, pharmaceutical compounds, and dyes.

Many researchers investigated the nature of the interaction between adsorbent and targeted contaminates via applying well-known adsorption isotherms such as Langmuir, Freundlich, Sips, Redlich–Peterson isotherm models (Ismael et al., 2016; Maged et al., 2021). Ge et al. (2015) stated that the isotherms results indicated that the Langmuir model provided a superior fit to the data than the Freundlich model when MK-based porous geopolymer sphere was utilized to absorb Cu(II). This implies that the adsorption of Cu (II) by conventional geopolymer will likely be a single-layer process. Lee et al. (2017) reported that removing Cs(I) by mesoporous geopolymer containing nanocrystalline zeolite happened through physisorption and chemisorption mechanisms, and was achieved through multiple layers of adsorption. Moreover, the adsorption of Cu(II) on metakaolin-sodium alginate hybrid geopolymer was facilitated by chemical bonding between Cu(II) and the utilized geopolymer (Ge et al., 2017). Conversely, Cd(II) adsorption on zeolite-based geopolymer was due to chemisorption (Javadian et al., 2015). The cationic dye removal observed in adsorption isotherms was attributed to the formation of a monolayer of chemical bonds on the surface of the geopolymer (Acisli et al., 2020). The findings of Jin et al. suggest that chemisorption is the mechanism controlling the methylene blue removal process, as evidenced by the ion exchange of Na^+^ present in the synthesized geopolymer and water in alkaline conditions, which exposed a high number of negative charges and was further confirmed by the pseudo-second-order kinetic model (Jin et al., 2021).

On the other hand, many authors used the characterization techniques such as FT-IR, SEM, EDX, and XRD to get insight into the adsorption mechanism at the microscopic level for various geopolymers. The wavelengths ranging from 400 to 4000 cm^−1^ were frequently utilized for FT-IR analysis. Jin et al. (2022) conducted the FT-IR analysis of MB dye, 3D printing geopolymer, and MB-loading geopolymer. The authors reported that the band at 1384.6 cm^−1^ belongs to the symmetric stretching vibration of C–N, indicating MB adsorption. The asymmetric band around 3581 cm^−1^ is ascribed to adsorbed water molecules. The observed shift in this band after the adsorption of MB and the subsequent increase in peak intensity can be attributed to the further adsorption of water molecules and the formation of hydrogen bonds between the nitrogen in the tertiary amine, silicon, and aluminum hydroxyls. Additionally, the lone pair electrons in [AlO4]5 − may form n-π interaction with delocalized π bonds in MB. The UV–Vis DRS has suggested the formation of an *n*-π interaction based on the emergence of an absorption band located at about 606 nm, which is likely due to *n* → π * electron transitions involving lone pair electrons in the [AlO4]^−5^ ion (Zhang et al., 2017) of 3D printed geopolymer (Jin et al., 2022).

### Comparative study for the performance of clay-based geopolymers

This comparative study was conducted to get insight into the performance of various clay-based geopolymers compared to commonly used adsorbents for similar adsorbates. In this study, the maximum adsorption capacity was the comparison tool (Tables 3 and 4). Overall, the results from this study showed that clay-based geopolymers have superior performance compared to commonly used adsorbents based on various material sources. Specifically, clay-based geopolymers were found in the recent literature to have a higher adsorption capacity, faster adsorption rate, and higher selectivity for specific adsorbates. In addition, clay-based geopolymer adsorbents have been found to be more stable under various environmental conditions and more resistant to chemical degradation. These findings indicate that clay-based geopolymers are a superior choice for adsorption applications.

**Table 3:** Summarizes the starting material for the utilized geopolymer, alkaline activator, targeted pollutant, and adsorption capacity (mg/g) of the most recent clay-based geopolymer published in the literature

Precursor	Alkaline activator	Targeted pollutant	Adsorption capacity (mg/g)	References
Bentonite	NaOH, Na_2_SiO_3_	Navy blue dye	6.34	(Khan et al., 2023)
Metakaolin		Mn(II)	72.34	(Kara et al., 2018)
Metakaolin, clinoptilolite		Cr(III)	21.84	(Andrejkovičová et al., 2016)
Metakaolin		Methylene Blue	43.48	(El Alouani et al., 2019)
Metakaolin		Tetracycline	20.45	(Al-husseiny & Ebrahim, 2022b)
Pyrophyllite clay	NaOH	Cd(II)	7.82	(Panda et al., 2018)
Pyrophyllite clay		Methylene blue	64.10	(Ettahiri et al., 2023)
Metakaolin		Pb(II)	45.10	(Tang et al., 2015)
Silica, metakaolin		Acid red 97	1814.27	(Netto et al., 2020)
Metakaolin, rice husk ash	KOH	Crystal Violet	276.90	(Barbosa et al., 2018)
Metakaolin	KOH, silica fume	Cu(II)	40.0	(Singhal et al., 2017)

**Table 4:** Performance evaluation of clay-based geopolymers compared to commonly used adsorbents for similar adsorbates in the literature

Adsorbent	Targeted pollutant	*Q*_max_ (mg/g)	References
*Heavy metals*			
Laterite clay-based geopolymer	Ni(II)	520.00	(Ghani et al., 2020)
Commercial activated carbon		185.18	(Karapınar, 2022)
Modified red mud/chitosan		31.66	(Luu et al., 2023)
Bacterial biomass (*Cystoseria indica*)		18.17	(Khajavian et al., 2019)
Metakaolin-based geopolymer	Pb(II)	519.50	(Wei et al., 2022)
Melon peel biochar		188.7	(Ozdes & Duran, 2021)
Intercalated kaolinite		46.45	(Maged et al., 2020b)
Metakaolin-based geopolymer	Zn(II)	74.53	(Kara et al., 2017)
Brewed tea waste		1.46	(Çelebi et al., 2020)
Metakaolin-based mesoporous geopolymer (organic modified)	Cu(II)	108.2	(Yu et al., 2020)
Melon peel biochar		106.4	(Ozdes & Duran, 2021)
Cow manure biochar		70.420	(Zhang et al., 2021)
Metakaolin-based geopolymer (mixed with coal fly ash)	Cd(II)	78.2	(Lan et al., 2020)
Metakaolin-based geopolymer (natural zeolite filler)		98.10	(Andrejkovičová et al., 2016)
Shanghai silty clay		8.90	(Wang & Zhang, 2021)
Metakaolin-based geopolymer	Mn(II)	72.34	(Kara et al., 2018)
*Spirodela polyrhiza* (L.) Schleiden		35.7	(Meitei & Prasad, 2014)
Thermal–chemical–modified rice husk		1.29	(Hossain et al., 2022)
*Dyes*			
Pyrophyllite clay–based geopolymer	Methylene blue	64.10	(Ettahiri et al., 2023)
Acid-treated *Plumeria alba*		45.45	(Deka et al., 2023)
Zeolite/activated carbon@MnO2 composite		67.56	(Shojaei & Esmaeili, 2022)
Metakaolin-based geopolymer (rice husk ash)	Methyl violet 10B	276.9	(Barbosa et al., 2018)
α-FeOOH@Luffa composite		243.9	(Diaf et al., 2022)
Modified fly ash via alkaline fusion		77.61	(Grassi et al., 2020)
Metakaolin-based geopolymer (magnetic/zero-valent iron)	Acid Red 97	1814.0	(Netto et al., 2020)
Macro-fungal (*Agaricus bisporus*) wastes		372.69	(Drumm et al., 2021)
*Agaricus bisporus* residue		352.15	(Aouaini et al., 2021)
Metakaolin-based geopolymer (rice husk silica, Fe_3_O_4_)	Acid green	400.00	(Rossatto et al., 2020)
Banyan (*Ficus benghalensis*) tree leaves		19.50	(Gul et al., 2023)
Organic–inorganic hybrid clay		397	(Thue et al., 2018)
*Pharmaceutical compounds*			
Metakaolin-based geopolymer microspheres (oleic acid modified)	Tetracycline	645.70	(Wang et al., 2022)
Fe/graphene		422.00	(Alatalo et al., 2019)
Activated bentonite		388.10	(Maged et al., 2020a)
Scenedesmus quadricauda		295.34	(Daneshvar et al., 2018)
Kaolinite-based geopolymer	5-Fluorouracil	329.70	(Abukhadra et al., 2022)
MCM-48/biopolymer composites		191.00	(Abukhadra et al., 2020)
Magadiite-CTAB		130.59	(Ge et al., 2019)

The laterite clay–based geopolymer possessed a superior sorption capacity (520.00 mg/g) for Ni(II) ions sorption (Ghani et al., 2020), compared with various adsorbents even commercial activated carbon (185.18 mg/g) (Karapınar, 2022). Also, the performance of clay-based geopolymer with evaluated via a comparison with various materials-based adsorbent such as calcined electroplating sludge waste (Peng et al., 2020), bentonite (Chang et al., 2020), powdered activated sludge (Aslan et al., 2018). Considering these adsorbents’ material and/or synthesis costs, clay-based geopolymers are the relatively best-selected sorbents material for eliminating heavy metals from an aqueous solution. In the case of dye removal, clay-based geopolymers showed a good performance; however, it was in the same adsorption capacities range of the other existing adsorbent. Nevertheless, the metakaolin-based geopolymer showed exceptional adsorption capacity for Acid Red 97 (1814.0 mg/g) and Acid green (400.00 mg/g) compared to the other sorbents for the literature (Netto et al., 2020; Rossatto et al., 2020). For pharmaceutical compounds, clay-based geopolymers demonstrated a high uptake capacity towards tetracycline and 5-fluorouracil compounds compared to the other synthesized or activated sorbent materials as presented in Table 4. However, not enough studies were available to comparatively evaluate clay-based geopolymers with other sorbents for the removal of pharmaceutical compounds from aqueous medium.

## Clays-based geopolymer role in climate change, SDGs, and circular economy

According to the United Nations Framework Convention on Climate Change (UNFCCC), the climate change phenomenon is commonly defined as a climate variation directly/indirectly caused by anthropogenic activities. Water pollution can have a direct and indirect impact on climate change. As pollutants are released into water bodies, they can slowly accumulate over time, increasing greenhouse gases in the atmosphere. This, in turn, can cause temperatures to rise, resulting in more frequent and intense weather events. Additionally, by depleting the water of oxygen and other essential nutrients, water pollution can lead to an imbalance in the local environment, further contributing to the effects of climate change. Dramatic climate change was demonstrated by a number of objective indicators that were documented over the past several centuries, including an increment in the air and ocean temperatures, a rise in sea level, glacier melting, growth in the frequency and strength of extreme weather events, changes in ocean salinity, wind patterns, and the annual distribution of rainfall, as well as an augmentation in the risks of hydrogeological hazards like floods, droughts, and fires (Abu El-Magd et al., 2022; Patel & Kuttippurath, 2021; Talukder et al., 2022). Such phenomena have negative undesirable impacts on anthropogenic, biological, and ecological systems including variations in the physiological responses, and alterations in the distribution assembly of different living species (e.g., migration, ecological communities change, geographical restructuring, population growth, and extinctions) (Amer et al., 2021; Fattorini, 2021; Gonçalves et al., 2021).

Water treatment from various existing pollutants is critical, as it helps protect public health and the environment. The existence of these pollutants in drinking water can lead to adverse health effects, so it is critical to have efficient and reliable water treatments in place. Furthermore, this process is directly linked to the sustainable development goals (SDGs). It contributes to achieving SDG 6.3 (Progress on Wastewater Treatment), which focuses on improving water quality and reducing water pollution. Treatment of pollutants from water can also help support SDG 3: Good Health and Well-Being, as it eliminates potential health risks associated with these contaminates. In addition, it can help reduce the burden on healthcare systems by minimizing the number of people affected by water-borne illnesses. Water treatment is also directly related to SDG 14: Life Below Water. Cleaning water from pollutants helps to reduce water pollution, which improves the health of aquatic ecosystems. It also helps to protect the biodiversity of marine and aquatic species and the habitats in which they live. We can protect our oceans and all the life they contain by ensuring that water is free from pollutants.

Over the past decades, the circular economy (CE) concept has gained popularity regarding traditional linear economies’ strains on natural resources. Besides the uncontrollable population growth and tremendous industrial progress, the conventional linear economy has exponentially consumed natural resources and produced massive waste (Sánchez Levoso et al., 2020). Using clay-based geopolymer in water treatment is an effective way to help the circular economy. Geopolymers are made from clay and other natural materials and can adsorb and neutralize pollutants, making them ideal for water treatment. Geopolymers also have a longer shelf life than other materials, making them a sustainable option for water treatment. Additionally, geopolymers are usually less expensive than other materials, making them an attractive choice for water treatment. Using clay-based geopolymer in water treatment is an effective way to reduce pollution and conserve resources, making it a viable option for helping to promote a circular economy.

Recently, the awareness of the environmental sustainability for wastewater treatment has increased rapidly in quest of meeting the enormous global water demand coupled with the inherent depletion of water resources and the development of modern society. The only practical approaches are new sustainable materials and products, green production techniques, and precise life cycle management. In this regard, clays-based geopolymers have emerged as affordable, durable, and eco-benevolent materials for water and wastewater clean-up. They can be synthesized via facile and straightforward strategies with minimal running temperatures and low carbon footprint yields. They exhibited remarkable physio-chemical characteristics of admirable porous structure, chemical stability, ion exchange, and dielectric features (Luhar et al., 2021). The exceptional clays-based geopolymers have significantly been touted as a keystone of wastewater treatment future entails. The integration of sustainable clay-based geopolymers in diverse industrial sectors should be harmonically considered. Moreover, these eco-friendly adsorbents are believed to deepen insights into wastewater treatment processes as a groundbreaking aspect in accord with the waste-to-wealth concept toward broader sustainable development goals.

## Challenges and future outlook

- The rapidly diminishing supply of clean water, the deterioration of water quality, and global warming significantly negatively influence the world’s water security and natural environment.
- This has prompted scientists globally to find new alternative eco-friendly wastewater treatment scenarios. Clays-based geopolymers have been regarded as favorable materials for wastewater reclamation.
- To achieve the SDGs, great efforts should be forwarded in this context for upcycling these abundant, low-cost materials into beneficial products. The fundamental essence is to present greater attention to the most popular wastewater reclamation methods to defeat their confronted challenges.
- Efforts should be made to develop more cost-effective synthesis methods, so that the use of clay-based geopolymer in water treatment can become more widespread.
- New research for the elimination of surfactants from aqueous solutions using clay-based geopolymer should be conducted as it has not yet been identified in the literature.
- Most experiments have only been conducted on a lab scale, making it evident that further research is required before using clays-based geopolymers as suitable materials for water decontamination.
- Additionally, it is essential to develop strategies for the safe disposal of clay-based geopolymers, as they are not biodegradable and can create environmental hazards if not disposed of properly.
- More mechanistic insight into recyclability/revalorization of the pollutants-laden materials is recommended because it is an important regulating factor for long-term economic viability and environmental compatibility.
- One of the potential future possibilities to assure their viability could be by integrating wastewater management with data-driven technologies such as machine learning, artificial neural networks, techno-economic analysis, and life cycle assessment for improving the process performance and simultaneously addressing better reusability and final product quality.
- Employing standard protocols with ongoing assessments for wastewater cleanup could be an effective solution for data quantification. Otherwise, the competence of clays-based geopolymers with other materials may potentially upgrade their activity towards different water pollutants. However, more studies should be economically inspected for large-scale industrial applications.
- Besides, The COVID-19 pandemic has also led to a rise in wastewater disposal. More attention should be paid to wastewater surveillance and recycling in line with the zero-liquid discharge concepts to realize a circular economy.
- Ultimately, assessing all the aspects related to clays-based geopolymers for wastewater treatment, including raw feedstock, synthesis routes, modes of action, and performance findings, is of interest for their development in wastewater treatment.

## Conclusion

Clays/clays-based geopolymers have emerged as admirable materials for wastewater treatment toward a more sustainable environment to meet the uncontrollable shortage in the availability of water resources and deterioration in their quality. Remarkably, they offer distinct physicochemical properties of excellent stability, large surface area, and long-term durability. The current review article uniquely illustrates the most recent significant information regarding clays/clays-based geopolymers, including the recent synthesis, modifications, and characterization strategies. They are regarded as alternative materials in accord with the sustainable circular economy concepts globally. The ingredients of clays and their based geopolymers have the potency to replace the conventional adsorbents currently employed in wastewater treatment. Economically, an adequate study of techno-economic analyses and life cycle assessment related to the commercial scaling-up of clays and their based geopolymers should be systematically inspected. Besides, a more in-depth analysis into the inexpensive clays/clays-based geopolymers manufacturing processes from natural resources should emphasize a more comprehensive future vision.

## Supplementary Information

Below is the link to the electronic supplementary material.

Supplementary file1 (DOCX 122 KB)

## Data Availability

The datasets generated during and/or analyzed during the current study are available from the corresponding author on reasonable request.

## Declarations

All authors have read, understood, and have complied as applicable with the statement on “Ethical responsibilities of Authors” as found in the Instructions for Authors.

## References

[CR1] Abafe OA, Späth J, Fick J, Jansson S, Buckley C, Stark A, Pietruschka B, Martincigh BS (2018). LC-MS/MS determination of antiretroviral drugs in influents and effluents from wastewater treatment plants in KwaZulu-Natal, South Africa. Chemosphere.

[CR2] Abd El-Fattah, H., Maged, A., Kamel, R. M., & Kharbish, S., (2023). Recent technologies for the elimination of pharmaceutical compounds from aqueous solutions: A review. *Frontiers in Food Science and Technology*, *5*, 0–0. 10.21608/FSRT.2023.173676.1074

[CR3] Abdollahizad G, Valadi FM, Akbarzadeh E, Gholami MR (2022). Adsorption properties of halloysite modified acrylamide/quince seeds-based hydrogel: Experimental and DFT investigation. Journal of Polymers and the Environment.

[CR4] Abu El-Magd SA, Maged A, Farhat HI (2022). Hybrid-based Bayesian algorithm and hydrologic indices for flash flood vulnerability assessment in coastal regions: Machine learning, risk prediction, and environmental impact. Environmental Science and Pollution Research.

[CR5] Abukhadra MR, Alhammadi AA, Khim JS, Ajarem JS, Allam AA, Shaban MS (2022). Enhanced adsorption and visible light photocatalytic removal of 5-fluorouracil residuals using environmental NiO/geopolymer nanocomposite: Steric, energetic, and oxidation studies. Journal of Environmental Chemical Engineering..

[CR6] Abukhadra MR, Refay NM, El-Sherbeeny AM, El-Meligy MA (2020). Insight into the loading and release properties of MCM-48/biopolymer composites as carriers for 5-fluorouracil: Equilibrium modeling and pharmacokinetic studies. ACS Omega.

[CR7] Acisli O, Acar I, Khataee A (2020). Preparation of a fly ash-based geopolymer for removal of a cationic dye: Isothermal, kinetic and thermodynamic studies. Journal of Industrial and Engineering Chemistry.

[CR8] Açışlı Ö, Acar İ, Khataee A (2022). Preparation of a surface modified fly ash-based geopolymer for removal of an anionic dye: Parameters and adsorption mechanism. Chemosphere.

[CR9] Adewuyi YG (2021). Recent advances in fly-ash-based geopolymers: Potential on the utilization for sustainable environmental remediation. ACS Omega.

[CR10] Al-Ghouti, M. A., Al-Degs, Y. S., Ghrair, A., Ziedan, M., Khoury, H., Abdelghani, J. I., & Khraisheh, M. (2021). Development of industrially viable geopolymers from treated petroleum fly ash. *Journal of Cleaner Production*, *280*. 10.1016/j.jclepro.2020.124808

[CR11] Al-Hussaini AS, Elias AM, Abd El-Ghaffar MA (2017). New poly(aniline-co-o-phenylenediamine)/kaolinite microcomposites for water decontamination. Journal of Polymers and the Environment.

[CR12] Al-husseiny RA, Ebrahim SE (2022). Synthesis of nano-magnetite and magnetite/synthetic geopolymer nano-porous composite for application as a novel adsorbent. Environmental Nanotechnology, Monitoring & Management..

[CR13] Al-husseiny, R. A., & Ebrahim, S. E. (2022b). Effective removal of methylene blue from wastewater using magnetite/geopolymer composite: Synthesis, characterization and column adsorption study. *Inorganic Chemistry Communications*, *139*. 10.1016/j.inoche.2022.109318

[CR14] Al Aukidy M, Verlicchi P, Jelic A, Petrovic M, Barcelò D (2012). Monitoring release of pharmaceutical compounds: Occurrence and environmental risk assessment of two WWTP effluents and their receiving bodies in the Po Valley, Italy. Science of the Total Environment.

[CR15] Alatalo S-MM, Daneshvar E, Kinnunen N, Meščeriakovas A, Thangaraj SK, Jänis J, Tsang DCW, Bhatnagar A, Lähde A (2019). Mechanistic insight into efficient removal of tetracycline from water by Fe/graphene. Chemical Engineering Journal.

[CR16] Amer O, Kharbish S, Maged A, Khedr F (2021). Geochemical insight into granite hosted U-rich fluorite, Gabal El-Erediya area, Central Eastern Desert, Egypt: REE geochemical and fluid inclusion aspects. Arabian Journal of Geosciences.

[CR17] Andrejkovičová S, Sudagar A, Rocha J, Patinha C, Hajjaji W, Da Silva EF, Velosa A, Rocha F (2016). The effect of natural zeolite on microstructure, mechanical and heavy metals adsorption properties of metakaolin based geopolymers. Applied Clay Science.

[CR18] Aouaini F, Sellaoui L, Alanazi MM, Dotto GL, Alfwzan W, Al-Yousef HA, Erto A (2021). Theoretical analysis of the removal mechanism of Crystal Violet and Acid Red 97 dyes on *Agaricus bisporus* residue. Journal of Molecular Liquids..

[CR19] Arokiasamy P, Abdullah MMAB, Abd Rahim SZ, Sadique M, Ming LY, Mohd Salleh MAA, Mohd Arif Zainol MRR, Ghazali CMR (2023). Diverse material based geopolymer towards heavy metals removal: A review. Journal of Materials Research and Technology.

[CR20] Aslan S, Yildiz S, Ozturk M (2018). Biosorption of Cu2+ and Ni2+ ions from aqueous solutions using waste dried activated sludge biomass. Polish Journal of Chemical Technology.

[CR21] Aydin S, Aydin ME, Ulvi A, Kilic H (2019). Antibiotics in hospital effluents: Occurrence, contribution to urban wastewater, removal in a wastewater treatment plant, and environmental risk assessment. Environmental Science and Pollution Research.

[CR22] Azuma T, Ishida M, Hisamatsu K, Yunoki A, Otomo K, Kunitou M, Shimizu M, Hosomaru K, Mikata S, Mino Y (2017). Fate of new three anti-influenza drugs and one prodrug in the water environment. Chemosphere.

[CR23] Barbosa TR, Foletto EL, Dotto GL, Jahn SL (2018). Preparation of mesoporous geopolymer using metakaolin and rice husk ash as synthesis precursors and its use as potential adsorbent to remove organic dye from aqueous solutions. Ceramics International.

[CR24] Bentahar Y, Hurel C, Draoui K, Khairoun S, Marmier N (2016). Adsorptive properties of Moroccan clays for the removal of arsenic(V) from aqueous solution. Applied Clay Science.

[CR25] Biel-Maeso M, Corada-Fernández C, Lara-Martín PA (2018). Monitoring the occurrence of pharmaceuticals in soils irrigated with reclaimed wastewater. Environmental Pollution.

[CR26] Bielecki, M., Brzoza-Brzezina, M., & Kolasa, M. (2020). Demographics and the natural interest rate in the euro area. *European Economic Review*, *129*. 10.1016/j.euroecorev.2020.103535

[CR27] Candamano S, Policicchio A, Conte G, Abarca R, Algieri C, Chakraborty S, Curcio S, Calabrò V, Crea F, Agostino RG (2022). Preparation of foamed and unfoamed geopolymer/NaX zeolite/activated carbon composites for CO_2_ adsorption. Journal of Cleaner Production.

[CR28] Çelebi H, Gök G, Gök O (2020). Adsorption capability of brewed tea waste in waters containing toxic lead(II), cadmium (II), nickel (II), and zinc(II) heavy metal ions. Science and Reports.

[CR29] Chang YS, Au PI, Mubarak NM, Khalid M, Jagadish P, Walvekar R, Abdullah EC (2020). Adsorption of Cu(II) and Ni(II) ions from wastewater onto bentonite and bentonite/GO composite. Environmental Science and Pollution Research.

[CR30] Chen, L., Wang, Z., Wang, Y., & Feng, J. (2016). Preparation and properties of alkali activated metakaolin-based geopolymer. *Materials (Basel)*, *9*. 10.3390/ma9090767 10.3390/ma9090767 PMC5457102 28773888

[CR31] Chen YL, Tong YY, Pan RW, Tang J (2013). The research on adsorption behaviors and mechanisms of geopolymers on Sr^2+^, Co^2+^ and Cs^+^. Advances in Materials Research.

[CR32] Chen Z, Wu G, Wu Y, Wu Q, Shi Q, Ngo HH, Vargas Saucedo OA, Hu HY (2020). Water Eco-Nexus Cycle System (WaterEcoNet) as a key solution for water shortage and water environment problems in urban areas. Water Cycle.

[CR33] Cheng TW, Lee ML, Ko MS, Ueng TH, Yang SF (2012). The heavy metal adsorption characteristics on metakaolin-based geopolymer. Applied Clay Science.

[CR34] Cong, P., & Cheng, Y.. (2021). Advances in geopolymer materials: A comprehensive review. *Journal of Traffic and Transportation Engineering (English Edition)*. 10.1016/j.jtte.2021.03.004

[CR35] Counts. (2023). Deaths from Dirty Water [WWW Document]. www.theworldcounts.com. URL https://www.theworldcounts.com/challenges/planet-earth/freshwater/deaths-from-dirty-water. Accessed 3.18.23.

[CR36] Dalstein F, Naqvi A (2022). 21st Century water withdrawal decoupling: A pathway to a more water-wise world?. Water Resources and Economics..

[CR37] Daneshvar E, Zarrinmehr MJ, Hashtjin AM, Farhadian O, Bhatnagar A (2018). Versatile applications of freshwater and marine water microalgae in dairy wastewater treatment, lipid extraction and tetracycline biosorption. Bioresource Technology.

[CR38] Davidovits, J. (2020). *Geopolymer chemistry and applications*, (5th ed.) J. Davidovits. Geopolymer Institute.

[CR39] de Ilurdoz MS, Sadhwani JJ, Reboso JV (2022). Antibiotic removal processes from water & wastewater for the protection of the aquatic environment - A review. Journal of Water Process Engineering.

[CR40] Deka J, Das H, Singh A, Barman P, Devi A, Bhattacharyya KG (2023). Methylene blue removal using raw and modified biomass *Plumeria alba* (white frangipani) in batch mode: Isotherm, kinetics, and thermodynamic studies. Environmental Monitoring and Assessment.

[CR41] Diaf R, Bendjeffal H, Djebli A, Mamine H, Metidji T, Bekakria H, Hattab Z, Bouhedja Y (2022). α-FeOOH@Luffa composite used as a cost-effective, robust, and eco-friendly adsorbent material to remove Methyl Violet 10B from water. Chemistry Africa.

[CR42] Drumm FC, Franco DSP, Georgin J, Grassi P, Jahn SL, Dotto GL (2021). Macro-fungal (*Agaricus bisporus*) wastes as an adsorbent in the removal of the acid red 97 and crystal violet dyes from ideal colored effluents. Environmental Science and Pollution Research.

[CR43] El Alouani M, Alehyen S, El Achouri M, Taibi M (2019). Preparation, characterization, and application of metakaolin-based geopolymer for removal of methylene blue from aqueous solution. Journal of Chemistry.

[CR44] El Alouani M, Saufi H, Moutaoukil G, Alehyen S, Nematollahi B, Belmaghraoui W, Taibi M (2021). Application of geopolymers for treatment of water contaminated with organic and inorganic pollutants: State-of-the-art review. Journal of Environmental Chemical Engineering.

[CR45] Elgarahy AM, Maged A, Elwakeel KZ, El-Gohary F, El-Qelish M (2023). Tuning cationic/anionic dyes sorption from aqueous solution onto green algal biomass for biohydrogen production. Environmental Research.

[CR46] Elsherbiny AS, El-Hefnawy ME, Gemeay AH (2018). Adsorption efficiency of polyaspartate-montmorillonite composite towards the removal of Pb(II) and Cd(II) from aqueous solution. Journal of Polymers and the Environment.

[CR47] Erdemoğlu M, Birinci M, Uysal T (2020). Thermal behavior of pyrophyllite ore during calcination for thermal activation for aluminum extraction by acid leaching. Clays and Clay Minerals.

[CR48] Ettahiri, Y., Bouna, L., Hanna, J. V., Benlhachemi, A., Pilsworth, H. L., Bouddouch, A., & Bakiz, B. (2023). Pyrophyllite clay-derived porous geopolymers for removal of methylene blue from aqueous solutions. *Materials Chemistry and Physics*, *296*. 10.1016/j.matchemphys.2022.127281

[CR49] Fattorini, S. (2021). Climate change and extinction events. In: Encyclopedia of Geology. 10.1016/b978-0-12-409548-9.12116-5

[CR50] Gamboni JE, Bertuzzi MA, Slavutsky AM (2022). Methylene blue sorption phenomena onto pectin, brea gum, montmorillonite based hydrogels: Kinetic and Thermodynamic assessment. Journal of Polymers and the Environment.

[CR51] Gao K, Lin KL, Wang D, Shiu HS, Hwang CL, Tuan BLA, Cheng TW (2014). Thin-film-transistor liquid-crystal display waste glass and nano-SiO 2 as substitute sources for metakaolin-based geopolymer. Environmental Progress & Sustainable Energy.

[CR52] Ge M, Tang W, Du M, Liang G, Hu G, Jahangir Alam SM (2019). Research on 5-fluorouracil as a drug carrier materials with its in vitro release properties on organic modified magadiite. European Journal of Pharmaceutical Sciences.

[CR53] Ge Y, Cui X, Liao C, Li Z (2017). Facile fabrication of green geopolymer/alginate hybrid spheres for efficient removal of Cu(II) in water: Batch and column studies. Chemical Engineering Journal.

[CR54] Ge Y, Yuan Y, Wang K, He Y, Cui X (2015). Preparation of geopolymer-based inorganic membrane for removing Ni2+ from wastewater. Journal of Hazardous Materials.

[CR55] Ghani U, Hussain S, Noor-ul-Amin, Imtiaz M, Ali Khan S (2020). Laterite clay-based geopolymer as a potential adsorbent for the heavy metals removal from aqueous solutions. Journal of Saudi Chemical Society.

[CR56] Gonçalves, F., Sales, L. P., Galetti, M., & Pires, M. M. (2021). Combined impacts of climate and land use change and the future restructuring of Neotropical bat biodiversity. *Perspectives in Ecology and Conservation*, *19*. 10.1016/j.pecon.2021.07.005

[CR57] Grassi P, Drumm FC, Franco DSP, Georgin J, Dotto GL, Foletto EL, Jahn SL (2020). Application of fly ash modified by alkaline fusion as an effective adsorbent to remove methyl violet 10B in water. Chemical Engineering Communications.

[CR58] Grim RE (1962). Applied clay mineralogy. GFF.

[CR59] Gul S, Gul A, Gul H, Khattak R, Ismail M, Khan SU, Khan MS, Aouissi HA, Krauklis A (2023). Removal of brilliant green dye from water using ficus benghalensis tree leaves as an efficient biosorbent. Materials (Basel)..

[CR60] Hajizadeh Z, Radinekiyan F, Eivazzadeh-keihan R, Maleki A (2020). Development of novel and green NiFe2O4/geopolymer nanocatalyst based on bentonite for synthesis of imidazole heterocycles by ultrasonic irradiations. Science and Reports.

[CR61] Hossain N, Nizamuddin S, Shah K (2022). Thermal-chemical modified rice husk-based porous adsorbents for Cu (II), Pb (II), Zn (II), Mn (II) and Fe (III) adsorption. Journal of Water Process Engineering.

[CR62] Hua P, Sellaoui L, Franco D, Netto MS, Luiz Dotto G, Bajahzar A, Belmabrouk H, Bonilla-Petriciolet A, Li Z (2020). Adsorption of acid green and procion red on a magnetic geopolymer based adsorbent: Experiments, characterization and theoretical treatment. Chemical Engineering Journal.

[CR63] Ismael IS, Kharbish S (2013). Removing of As (V) from aqueous solution using natural and pretreated glauconite and halloysite. Carpathian Journal of Earth and Environmental Sciences.

[CR64] Ismael IS, Kharbish S, Saad EM, Maged A (2016). Adsorption of copper from aqueous solutions by using natural clay. Acta Universitatis Matthiae BELII: Séria Environmentálne Manažérstvo.

[CR65] Jahan I, Zhang L (2022). Natural polymer-based electrospun nanofibrous membranes for wastewater treatment: A review. Journal of Polymers and the Environment.

[CR66] Javadian H, Ghorbani F, Tayebi H, Asl SH (2015). Study of the adsorption of Cd (II) from aqueous solution using zeolite-based geopolymer, synthesized from coal fly ash; Kinetic, isotherm and thermodynamic studies. Arabian Journal of Chemistry.

[CR67] Jin H, Zhang Y, Wang Q, Chang Q, Li C (2021). Rapid removal of methylene blue and nickel ions and adsorption/desorption mechanism based on geopolymer adsorbent. Colloid and Interface Science Communications.

[CR68] Jin H, Zhang Y, Zhang X, Chang M, Li C, Lu X, Wang Q (2022). 3D printed geopolymer adsorption sieve for removal of methylene blue and adsorption mechanism. Colloids and Surfaces A: Physicochemical and Engineering Aspects.

[CR69] Kara I, Tunc D, Sayin F, Akar ST (2018). Study on the performance of metakaolin based geopolymer for Mn(II) and Co(II) removal. Applied Clay Science.

[CR70] Kara İ, Yilmazer D, Akar ST (2017). Metakaolin based geopolymer as an effective adsorbent for adsorption of zinc(II) and nickel(II) ions from aqueous solutions. Applied Clay Science.

[CR71] Karapınar HS (2022). Adsorption performance of activated carbon synthesis by ZnCl2, KOH, H3PO4 with different activation temperatures from mixed fruit seeds. Environmental Technology (United Kingdom).

[CR72] Khajavian M, Wood DA, Hallajsani A, Majidian N (2019). Simultaneous biosorption of nickel and cadmium by the brown algae *Cystoseria indica* characterized by isotherm and kinetic models. Applied Biological Chemistry.

[CR73] Khale D, Chaudhary R (2007). Mechanism of geopolymerization and factors influencing its development: A review. Journal of Materials Science.

[CR74] Khan H, Hussain S, Zahoor R, Arshad M, Umar M, Marwat MA, Khan A, Khan JR, Haleem MA (2023). Novel modeling and optimization framework for Navy Blue adsorption onto eco-friendly magnetic geopolymer composite. Environmental Research.

[CR75] Khan MI, Min TK, Azizli K, Sufian S, Ullah H, Man Z (2015). Effective removal of methylene blue from water using phosphoric acid based geopolymers: Synthesis, characterizations and adsorption studies. RSC Advances.

[CR76] Korkmaz, N. E., Caglar, N. B., & Aksu, A. (2022). Presence and distribution of selected pharmaceutical compounds in water and surface sediment of the Golden Horn Estuary, Sea of Marmara, Turkey. *Regional Studies in Marine Science*, *51*. 10.1016/j.rsma.2022.102221

[CR77] Kumar A, Subrahmanyam G, Mondal R, Cabral-Pinto MMS, Shabnam AA, Jigyasu DK, Malyan SK, Fagodiya RK, Khan SA, Yu ZG (2021). Bio-remediation approaches for alleviation of cadmium contamination in natural resources. Chemosphere.

[CR78] Lan T, Guo S, Li X, Guo J, Bai T, Zhao Q, Yang W, Li P (2020). Mixed precursor geopolymer synthesis for removal of Pb(II) and Cd(II). Materials Letters.

[CR79] Lazaratou CV, Vayenas DV, Papoulis D (2020). The role of clays, clay minerals and clay-based materials for nitrate removal from water systems: A review. Applied Clay Science.

[CR80] Lee NK, Khalid HR, Lee HK (2017). Adsorption characteristics of cesium onto mesoporous geopolymers containing nano-crystalline zeolites. Microporous and Mesoporous Materials.

[CR81] Lemougna PN, Wang K, Tang Q, Melo UC, Cui X (2016). Recent developments on inorganic polymers synthesis and applications. Ceramics International.

[CR82] Liang K, Wang XQ, Chow CL, Lau D (2022). A review of geopolymer and its adsorption capacity with molecular insights: A promising adsorbent of heavy metal ions. Journal of Environmental Management.

[CR83] Liew YM, Heah CY, Mohd Mustafa AB, Kamarudin H (2016). Structure and properties of clay-based geopolymer cements: A review. Progress in Materials Science.

[CR84] Liu Y, Yan C, Qiu X, Li D, Wang H, Alshameri A (2016). Preparation of faujasite block from fly ash-based geopolymer via in-situ hydrothermal method. Journal of the Taiwan Institute of Chemical Engineers.

[CR85] Luhar I, Luhar S (2021). Rubberized geopolymer composites: Value-added applications. Journal of Composites Science.

[CR86] Luhar I, Luhar S, Abdullah MMAB, Razak RA, Vizureanu P, Sandu AV, Matasaru PD (2021). A state-of-the-art review on innovative geopolymer composites designed for water and wastewater treatment. Materials (Basel).

[CR87] Luu TT, Nguyen DK, Nguyen TTP, Ho TH, Dinh VP, Kiet HAT (2023). The effective Ni(II) removal of red mud modified chitosan from aqueous solution. Environmental Monitoring and Assessment.

[CR88] Luukkonen T, Runtti H, Niskanen M, Tolonen E-T, Sarkkinen M, Kemppainen K, Rämö J, Lassi U (2016). Simultaneous removal of Ni(II), As(III), and Sb(III) from spiked mine effluent with metakaolin and blast-furnace-slag geopolymers. Journal of Environmental Management.

[CR89] Maged A, Abu El-Magd SA, Radwan AE, Kharbish S, Zamzam S (2023). Evaluation insight into Abu Zenima clay deposits as a prospective raw material source for ceramics industry: Remote sensing and characterization. Science and Reports.

[CR90] Maged A, Dissanayake PD, Yang X, Pathirannahalage C, Bhatnagar A, Ok YS (2021). New mechanistic insight into rapid adsorption of pharmaceuticals from water utilizing activated biochar. Environmetal Research.

[CR91] Maged A, Iqbal J, Kharbish S, Ismael ISIS, Bhatnagar A (2020). Tuning tetracycline removal from aqueous solution onto activated 2:1 layered clay mineral: Characterization, sorption and mechanistic studies. Journal of Hazardous Materials.

[CR92] Maged A, Ismael IS, Kharbish S, Sarkar B, Peräniemi S, Bhatnagar A (2020). Enhanced interlayer trapping of Pb(II) ions within kaolinite layers: Intercalation, characterization, and sorption studies. Environmental Science and Pollution Research.

[CR93] Maged A, Kharbish S, Ismael ISIS, Bhatnagar A (2020). Characterization of activated bentonite clay mineral and the mechanisms underlying its sorption for ciprofloxacin from aqueous solution. Environmental Science and Pollution Research.

[CR94] Mahmood AR, Al-Haideri HH, Hassan FM (2019). Detection of antibiotics in drinking water treatment plants in Baghdad City, Iraq. Advances in Public Health.

[CR95] Maleki A, Hajizadeh Z, Sharifi V, Emdadi Z (2019). A green, porous and eco-friendly magnetic geopolymer adsorbent for heavy metals removal from aqueous solutions. Journal of Cleaner Production.

[CR96] Malima, N. M., Owonubi, S. J., Lugwisha, E. H., & Mwakaboko, A. S. (2021). Development of cost-effective and eco-friendly adsorbent by direct physical activation of Tanzanian Malangali kaolinite for efficient removal of heavy metals. In: *Materials Today: Proceedings* (pp. 1126–1132). Elsevier. 10.1016/j.matpr.2020.06.469

[CR97] Meitei MD, Prasad MNV (2014). Adsorption of Cu (II), Mn (II) and Zn (II) by *Spirodela polyrhiza* (L.) Schleiden: Equilibrium, kinetic and thermodynamic studies. Ecological Engineering.

[CR98] Morales-Paredes CA, Rodríguez-Díaz JM, Boluda-Botella N (2022). Pharmaceutical compounds used in the COVID-19 pandemic: A review of their presence in water and treatment techniques for their elimination. Science of the Total Environment.

[CR99] Netto MS, Leindcker Rossatto D, Jahn SL, Stoffels Mallmann E, Luiz Dotto G, Luiz Foletto E (2020). Preparation of a novel magnetic geopolymer/zero–valent iron composite with remarkable adsorption performance towards aqueous Acid Red 97. Chemical Engineering Communications.

[CR100] Novais RM, Ascensão G, Tobaldi DM, Seabra MP, Labrincha JA (2018). Biomass fly ash geopolymer monoliths for effective methylene blue removal from wastewaters. Journal of Cleaner Production.

[CR101] Novais RM, Buruberri LH, Seabra MP, Labrincha JA (2016). Novel porous fly-ash containing geopolymer monoliths for lead adsorption from wastewaters. Journal of Hazardous Materials.

[CR102] Novais RM, Carvalheiras J, Tobaldi DM, Seabra MP, Pullar RC, Labrincha JA (2019). Synthesis of porous biomass fly ash-based geopolymer spheres for efficient removal of methylene blue from wastewaters. Journal of Cleaner Production.

[CR103] Olatunde JO, Chimezie A, Tolulope B, Aminat TT (2014). Determination of pharmaceutical compounds in surface and underground water by solid phase extraction-liquid chromatography. Journal of Environmental Chemistry and Ecotoxicology.

[CR104] Otunola BO, Ololade OO (2020). A review on the application of clay minerals as heavy metal adsorbents for remediation purposes. Environmental Technology & Innovation.

[CR105] Ozdes D, Duran C (2021). Preparation of melon peel biochar/CoFe2O4 as a new adsorbent for the separation and preconcentration of Cu(II), Cd(II), and Pb(II) ions by solid-phase extraction in water and vegetable samples. Environmental Monitoring and Assessment.

[CR106] Padmapriya M, Ramesh ST, Biju VM (2022). Synthesis of seawater based geopolymer: Characterization and adsorption capacity of methylene blue from wastewater. Materials Today: Proceedings.

[CR107] Panda L, Rath SS, Rao DS, Nayak BB, Das B, Misra PK (2018). Thorough understanding of the kinetics and mechanism of heavy metal adsorption onto a pyrophyllite mine waste based geopolymer. Journal of Molecular Liquids.

[CR108] Papageorgiou, M., Zioris, I., Danis, T., Bikiaris, D., & Lambropoulou, D. (2019). Comprehensive investigation of a wide range of pharmaceuticals and personal care products in urban and hospital wastewaters in Greece. *Science of the Total Environment*, *694*. 10.1016/j.scitotenv.2019.07.371 10.1016/j.scitotenv.2019.07.371 31401503

[CR109] Patel, V. K., & Kuttippurath, J. (2021). Significant Increase in the water vapour over India and Indian Ocean: Implications for tropospheric warming and regional climate forcing. *SSRN Electronic Journal*. 10.2139/ssrn.3967422 10.1016/j.scitotenv.2022.155885 35595133

[CR110] Peng, G., Deng, S., Liu, F., Li, T., & Yu, G. (2020). Superhigh adsorption of nickel from electroplating wastewater by raw and calcined electroplating sludge waste. *Journal of Cleaner Production*, *246*. 10.1016/j.jclepro.2019.118948

[CR111] Pimraksa K, Setthaya N, Thala M, Chindaprasirt P, Murayama M (2020). Geopolymer/zeolite composite materials with adsorptive and photocatalytic properties for dye removal. PLoS One.

[CR112] Pourabbas Bilondi M, Toufigh MM, Toufigh V (2018). Experimental investigation of using a recycled glass powder-based geopolymer to improve the mechanical behavior of clay soils. Construction and Building Materials.

[CR113] Price JI, Heberling MT (2018). The Effects of source water quality on drinking water treatment costs: A review and synthesis of empirical literature. Ecological Economics.

[CR114] Rahier H, Wastiels J, Biesemans M, Willlem R, Van Assche G, Van Mele B (2007). Reaction mechanism, kinetics and high temperature transformations of geopolymers. Journal of Materials Science.

[CR115] Rasaki, S. A., Bingxue, Z., Guarecuco, R., Thomas, T., & Minghui, Y. (2019). Geopolymer for use in heavy metals adsorption, and advanced oxidative processes: A critical review. *Journal of Cleaner Production*. 10.1016/j.jclepro.2018.12.145 (Elsevier)

[CR116] Ren B, Zhao Y, Bai H, Kang S, Zhang T, Song S (2021). Eco-friendly geopolymer prepared from solid wastes: A critical review. Chemosphere.

[CR117] Rodriguez-Gil JL, San Sebastián Sauto J, González-Alonso S, Sánchez Sanchez P, Valcarcel Y, Catalá M (2013). Development of cost-effective strategies for environmental monitoring of irrigated areas in Mediterranean regions: Traditional and new approaches in a changing world. Agriculture, Ecosystems & Environment.

[CR118] Rossatto DL, Netto MS, Jahn SL, Mallmann ES, Dotto GL, Foletto EL (2020). Highly efficient adsorption performance of a novel magnetic geopolymer/Fe3O4 composite towards removal of aqueous acid green 16 dye. Journal of Environmental Chemical Engineering.

[CR119] Rowney NC, Johnson AC, Williams RJ (2011). Erratum: Cytotoxic drugs in drinking water: A prediction and risk assessment exercise for the Thames catchment in the United Kingdom. Environmental Toxicology and Chemistry.

[CR120] Rożek P, Król M, Mozgawa W (2018). Spectroscopic studies of fly ash-based geopolymers. Spectrochimica Acta, Part A: Molecular and Biomolecular Spectroscopy.

[CR121] Salam MA, Mokhtar M, Albukhari SM, Baamer DF, Palmisano L, Abukhadra MR (2021). Insight into the role of the zeolitization process in enhancing the adsorption performance of kaolinite/diatomite geopolymer for effective retention of Sr (II) ions; batch and column studies. Journal of Environmental Management.

[CR122] Salih SS, Kadhom M, Shihab MA, Ghosh TK (2022). Competitive adsorption of Pb(II) and phenol onto modified chitosan/vermiculite adsorbents. Journal of Polymers and the Environment.

[CR123] Samara E, Matsi T, Zdragas A, Barbayiannis N (2019). Use of clay minerals for sewage sludge stabilization and a preliminary assessment of the treated sludge’s fertilization capacity. Environmental Science and Pollution Research.

[CR124] Sánchez Levoso, A., Gasol, C. M., Martínez-Blanco, J., Durany, X. G., Lehmann, M., & Gaya, R. F. (2020). Methodological framework for the implementation of circular economy in urban systems. *Journal of Cleaner Production*, *248*. 10.1016/j.jclepro.2019.119227

[CR125] Sarkar C, Basu JK, Samanta AN (2017). Removal of Ni2+ ion from waste water by geopolymeric adsorbent derived from LD Slag. Journal of Water Process Engineering.

[CR126] Sdiri A, Khairy M, Bouaziz S, El-Safty S (2016). A natural clayey adsorbent for selective removal of lead from aqueous solutions. Applied Clay Science.

[CR127] Shojaei M, Esmaeili H (2022). Ultrasonic-assisted synthesis of zeolite/activated carbon@MnO2 composite as a novel adsorbent for treatment of wastewater containing methylene blue and brilliant blue. Environmental Monitoring and Assessment.

[CR128] Singh B, Ishwarya G, Gupta M, Bhattacharyya SK (2015). Geopolymer concrete: A review of some recent developments. Construction and Building Materials.

[CR129] Singhal A, Gangwar BP, Gayathry JM (2017). CTAB modified large surface area nanoporous geopolymer with high adsorption capacity for copper ion removal. Applied Clay Science.

[CR130] Siyal AA, Shamsuddin MR, Khahro SH, Low A, Ayoub M (2021). Optimization of synthesis of geopolymer adsorbent for the effective removal of anionic surfactant from aqueous solution. Journal of Environmental Chemical Engineering.

[CR131] Siyal AA, Shamsuddin MR, Khan MI, Rabat NE, Zulfiqar M, Man Z, Siame J, Azizli KA (2018). A review on geopolymers as emerging materials for the adsorption of heavy metals and dyes. Journal of Environmental Management.

[CR132] Siyal AA, Shamsuddin MR, Rabat NE, Zulfiqar M, Man Z, Low A (2019). Fly ash based geopolymer for the adsorption of anionic surfactant from aqueous solution. Journal of Cleaner Production.

[CR133] Strozi Cilla M, Raymundo Morelli M, Colombo P (2014). Effect of process parameters on the physical properties of porous geopolymers obtained by gelcasting. Ceramics International.

[CR134] Tahmasebi Yamchelou, M., Law, D., Brkljača, R., Gunasekara, C., Li, J., & Patnaikuni, I. (2021). Geopolymer synthesis using low-grade clays. *Construction and Building Materials*, *268*. 10.1016/j.conbuildmat.2020.121066

[CR135] Talukder, B., Ganguli, N., Matthew, R., vanLoon, G. W., Hipel, K. W., & Orbinski, J. (2022). Climate change-accelerated ocean biodiversity loss & associated planetary health impacts. *The Journal of Climate Change and Health*, *6*. 10.1016/j.joclim.2022.100114

[CR136] Tan TH, Mo KH, Lai SH, Ling TC (2021). Synthesis of porous geopolymer sphere for Ni(II) removal. Ceramics International.

[CR137] Tan, T. H., Mo, K. H., Ling, T. C., & Lai, S. H. (2020). Current development of geopolymer as alternative adsorbent for heavy metal removal. *Environmental Technology & Innovation*. 10.1016/j.eti.2020.100684

[CR138] Tang Q, Ge Y, Wang K, He Y, Cui X (2015). Preparation and characterization of porous metakaolin-based inorganic polymer spheres as an adsorbent. Materials and Design.

[CR139] The World Counts. (2023). Deaths from dirty water [WWW Document]. The World Counts. URL https://www.theworldcounts.com/challenges/planet-earth/freshwater/deaths-from-dirty-water. Accessed 2.3.23.

[CR140] Thue PS, Sophia AC, Lima EC, Wamba AGN, de Alencar WS, dos Reis GS, Rodembusch FS, Dias SLP (2018). Synthesis and characterization of a novel organic-inorganic hybrid clay adsorbent for the removal of acid red 1 and acid green 25 from aqueous solutions. Journal of Cleaner Production.

[CR141] Tian Q, Bai Y, Pan Y, Chen C, Yao S, Sasaki K, Zhang H (2022). Application of geopolymer in stabilization/solidification of hazardous pollutants: A review. Molecules.

[CR142] Tochetto GA, Simão L, de Oliveira D, Hotza D, Immich APS (2022). Porous geopolymers as dye adsorbents: Review and perspectives. Journal of Cleaner Production.

[CR143] Tran ML, Tran TTV, Juang RS, Nguyen CH (2023). Graphene oxide crosslinked chitosan composites for enhanced adsorption of cationic dye from aqueous solutions. Journal of the Taiwan Institute of Chemical Engineers.

[CR144] Tran TTV, Kumar SR, Lue SJ (2019). Separation mechanisms of binary dye mixtures using a PVDF ultrafiltration membrane: Donnan effect and intermolecular interaction. Journal of Membrane Science.

[CR145] Uddin MJ, Ampiaw RE, Lee W (2021). Adsorptive removal of dyes from wastewater using a metal-organic framework: A review. Chemosphere.

[CR146] Uddin MK (2017). A review on the adsorption of heavy metals by clay minerals, with special focus on the past decade. Chemical Engineering Journal.

[CR147] Usman ARA, Kuzyakov Y, Stahr K (2004). Effect of clay minerals on extractability of heavy metals and sewage sludge mineralization in soil. Chemical Ecology.

[CR148] Verma S, Amritphale SS, Khan MA, Anshul A, Das S (2017). Development of advanced geopolymerized brine sludge based composites. Journal of Polymers and the Environment.

[CR149] Vhahangwele M, Mugera GW (2015). The potential of ball-milled South African bentonite clay for attenuation of heavy metals from acidic wastewaters: Simultaneous sorption of Co2+, Cu2+, Ni2+, Pb2+, and Zn2+ ions. Journal of Environmental Chemical Engineering.

[CR150] Wahba, M. M., Labib, B., Darwish, K. M., & Zaghloul, M. (2017). Application of some clay minerals to eliminate the hazards of heavy metals in contaminated soils. 15th Int. Conf. Environ. Sci. Technol. 2015–2018.

[CR151] Wan Q, Rao F, Song S (2017). Reexamining calcination of kaolinite for the synthesis of metakaolin geopolymers - roles of dehydroxylation and recrystallization. Journal of Non-Crystalline Solids.

[CR152] Wang C, Yang Z, Song W, Zhong Y, Sun M, Gan T, Bao B (2021). Quantifying gel properties of industrial waste-based geopolymers and their application in Pb2+ and Cu2+ removal. Journal of Cleaner Production.

[CR153] Wang G, Ran L, Xu J, Wang Y, Ma L, Zhu R, Wei J, He H, Xi Y, Zhu J (2021). Technical development of characterization methods provides insights into clay mineral-water interactions: A comprehensive review. Applied Clay Science.

[CR154] Wang J, Zhang D, Liu S, Wang C (2020). Enhanced removal of chromium(III) for aqueous solution by EDTA modified attapulgite: Adsorption performance and mechanism. Science of the Total Environment.

[CR155] Wang J, Zhang W (2021). Evaluating the adsorption of Shanghai silty clay to Cd(II), Pb(II), As(V), and Cr(VI): Kinetic, equilibrium, and thermodynamic studies. Environmental Monitoring and Assessment.

[CR156] Wang X, Zhang Z, Ge Y (2022). Oleic acid-tailored geopolymer microspheres with tunable porous structure for enhanced removal from tetracycline in saline water. Sustainability.

[CR157] Wei E, Wang K, Muhammad Y, Chen S, Dong D, Wei Y, Fujita T (2022). Preparation and conversion mechanism of different geopolymer-based zeolite microspheres and their adsorption properties for Pb2+. Separation and Purification Technology.

[CR158] WHO. (2022). Drinking-water [WWW Document]. URL https://www.who.int/news-room/fact-sheets/detail/drinking-water. Accessed 2.3.23.

[CR159] Wood TP, Duvenage CSJ, Rohwer E (2015). The occurrence of anti-retroviral compounds used for HIV treatment in South African surface water. Environmental Pollution.

[CR160] Wormington AM, De María M, Kurita HG, Bisesi JH, Denslow ND, Martyniuk CJ (2020). Antineoplastic agents: Environmental prevalence and adverse outcomes in aquatic organisms. Environmental Toxicology and Chemistry.

[CR161] Xue Q, Wang R, Liu S, Shi W, Tong X, Li Y, Sun F (2021). Significance of chlorite hyperspectral and geochemical characteristics in exploration: A case study of the giant Qulong porphyry Cu-Mo deposit in collisional orogen, Southern Tibet. Ore Geology Reviews.

[CR162] Yan C, Guo L, Ren D, Duan P (2019). Novel composites based on geopolymer for removal of Pb(II). Materials Letters.

[CR163] Yan S, Zhang F, Wang L, Rong Y, He P, Jia D, Yang J (2019). A green and low-cost hollow gangue microsphere/geopolymer adsorbent for the effective removal of heavy metals from wastewaters. Journal of Environmental Management.

[CR164] Yang, D., Yang, Y., & Xia, J. (2021). Hydrological cycle and water resources in a changing world: A review. *Geogre Sustain*. 10.1016/j.geosus.2021.05.003

[CR165] Yu Z, Song W, Li J, Li Q (2020). Improved simultaneous adsorption of Cu(II) and Cr(VI) of organic modified metakaolin-based geopolymer. Arabian Journal of Chemistry.

[CR166] Zhang G, Savateev A, Zhao Y, Li L, Antonietti M (2017). Advancing the n → π∗ electron transition of carbon nitride nanotubes for H2 photosynthesis. Journal of Materials Chemistry A.

[CR167] Zhang P, Zhang X, Yuan X, Xie R, Han L (2021). Characteristics, adsorption behaviors, Cu(II) adsorption mechanisms by cow manure biochar derived at various pyrolysis temperatures. Bioresource Technology.

[CR168] Zhang Y, Xiao R, Jiang X, Li W, Zhu X, Huang B (2020). Effect of particle size and curing temperature on mechanical and microstructural properties of waste glass-slag-based and waste glass-fly ash-based geopolymers. Journal of Cleaner Production.

[CR169] Zhang YJ, Yang MY, Zhang L, Zhang K, Kang L (2016). A new graphene/geopolymer nanocomposite for degradation of dye wastewater. Integrated Ferroelectronics.

[CR170] Zotiadis V, Argyraki A (2017). Development of innovative environmental applications of attapulgite clay. Bulletin Geological Society of Greece.

