# Supplementary material for: A comprehensive review on sustainable clay-based geopolymers for wastewater treatment: circular economy and future outlook
Source: Environ Monit Assess. 2023 May 19;195(6):693. doi: 10.1007/s10661-023-11303-9 (PMC10197063; doi:10.1007/s10661-023-11303-9)
Supplement: Supplementary file 1 — Supplementary file1 (DOCX 122 KB) [file 10661_2023_11303_MOESM1_ESM.docx]

A comprehensive review on sustainable clay based geopolymers for wastewater treatment: circular economy and future outlook

Ali Maged^1,*^, Hadeer Abd El-Fattah^2^, Rasha M. Kamel^2^, Sherif Kharbish^1^, Ahmed M. Elgarahy^3,4^

^1^ *Geology Department, Faculty of Science, Suez University, El Salam City, P.O. Box 43518, Suez Governorate, Egypt*

^2^ *Chemistry Department, Faculty of Science, Suez University, El Salam City, P.O. Box 43518, Suez Governorate, Egypt*

^3^ *Egyptian propylene and polypropylene company (EPPC), Port-Said, Egypt.*

^4^ *Environmental Chemistry Division, Environmental Science Department, Faculty of Science, Port Said University, Port Said, Egypt*

*Corresponding author: Ali Maged ([Ali.Maged@suezuni.edu.eg](mailto:Ali.Maged@suezuni.edu.eg))

Orcid: 0000-0001-5257-8523

**Figure S1.** Concentration of major anti-COVID-19 drugs in surface water and domestic wastewater before and during the pandemic, reused with permission from Elsevier (Morales-Paredes et al. 2022).

**References**

Morales-Paredes CA, Rodríguez-Díaz JM, Boluda-Botella N (2022) Pharmaceutical compounds used in the COVID-19 pandemic: A review of their presence in water and treatment techniques for their elimination. Sci Total Environ 814:152691. https://doi.org/10.1016/j.scitotenv.2021.152691
